# Progress towards the 2020 milestones of the end TB strategy in Cambodia: estimates of age and sex specific TB incidence and mortality from the Global Burden of Disease Study 2019

**DOI:** 10.1186/s12879-022-07891-5

**Published:** 2022-12-03

**Authors:** Jianing Ma, Avina Vongpradith, Jorge R. Ledesma, Amanda Novotney, Siyan Yi, Kruy Lim, Simon I. Hay, Christopher J. L. Murray, Hmwe H. Kyu

**Affiliations:** 1 Institute for Health Metrics and Evaluation, University of Washington, 3980 15Th Ave. NE, Seattle, WA 98195 USA; 2 Department of Health Metrics Sciences, University of Washington, Seattle, WA USA; 3 Saw Swee Hock School of Public Health, National University of Singapore and National University Health System, Singapore, Singapore; 4 KHANA Center for Population Health Research, Phnom Penh, Cambodia; 5 Center for Global Health Research, Public Health Program, Touro University California, Vallejo, CA USA; 6 Sihanouk Hospital Center of Hope, Phnom Penh, Cambodia

**Keywords:** Tuberculosis burden, Cambodia, GBD study, Risk factors, HIV-TB

## Abstract

**Background:**

Cambodia was recently removed from the World Health Organization’s (WHO’s) top 30 high tuberculosis (TB) burden countries. However, Cambodia’s TB burden remains substantial, and the country is on the WHO’s new global TB watchlist. We aimed to examine the levels and trends in the fatal and non-fatal TB burden in Cambodia from 1990 to 2019, assessing progress towards the WHO End TB interim milestones, which aim to reduce TB incidence rate by 20% and TB deaths by 35% from 2015 to 2020.

**Methods:**

We leveraged the Global Burden of Disease 2019 (GBD 2019) analytical framework to compute age- and sex-specific TB mortality and incidence by HIV status in Cambodia. We enumerated TB mortality utilizing a Bayesian hierarchical Cause of Death Ensemble modeling platform. We analyzed all available data sources, including prevalence surveys, population-based tuberculin surveys, and TB cause-specific mortality, to produce internally consistent estimates of incidence and mortality using a compartmental meta-regression tool (DisMod-MR 2.1). We further estimated the fraction of tuberculosis mortality among individuals without HIV coinfection attributable to the independent effects of alcohol use, smoking, and diabetes.

**Results:**

In 2019, there were 6500 (95% uncertainty interval 4830–8680) deaths due to all-form TB and 50.0 (43.8–57.8) thousand all-form TB incident cases in Cambodia. The corresponding age-standardized rates were 53.3 (39.9–69.4) per 100,000 population for mortality and 330.5 (289.0–378.6) per 100,000 population for incidence. From 2015 to 2019, the number of all-form TB deaths decreased by 11.8% (2.3–21.1), while the age-standardized all-form TB incidence rate decreased by 11.1% (6.3–15.6). Among individuals without HIV coinfection in 2019, alcohol use accounted for 28.1% (18.2–37.9) of TB deaths, smoking accounted for 27.0% (20.2–33.3), and diabetes accounted for 12.5% (7.1–19.0). Removing the combined effects of these risk factors would reduce all-form TB deaths by 54.2% (44.2–62.2).

**Discussion:**

Despite significant progress in reducing TB morbidity and mortality since 1990, Cambodia is not on track to achieve the 2020 WHO End TB interim milestones. Existing programs in Cambodia can benefit from liaising with risk factor control initiatives to accelerate progress toward eliminating TB in Cambodia.

**Supplementary Information:**

The online version contains supplementary material available at 10.1186/s12879-022-07891-5.

## Background

Despite being a preventable and treatable disease, tuberculosis (TB) remains a major global health challenge and is a leading infectious cause of death from a single agent [1, 2]. TB disease burden is disproportionately concentrated in low-income and middle-income countries (LMICs), where 90–95 percent of all TB deaths occur [3, 4]. LMICs confront substantial challenges in controlling TB, including: limited health care access, inadequate TB diagnostic capabilities, and TB social stigma contributing to poor TB detection and delayed initiation of treatment [5–8]. High economic costs associated with TB treatment in LMICs further exacerbate suboptimal treatment uptake [9–11]. Moreover, poor socio-economic conditions such as poor ventilation, overcrowding, and high HIV prevalence exacerbate TB transmission [12–14]. These challenges illustrate an urgent need for understanding TB trends in low-resource settings to monitor and evaluate the progress of key policies and programs.

Cambodia was recently removed from the World Health Organization’s (WHO’s) top 30 high TB burden countries. However, Cambodia’s TB burden remains high, and the country remains on the WHO’s new global TB watchlist [4]. Cambodia’s last national TB prevalence survey, conducted in 2011, found an active TB prevalence of 817 (95% confidence interval 690–954) per 100,000 population [15]. While the study showed that active TB prevalence has declined substantially since the previous national TB prevalence survey in 2002, the prevalence survey revealed that Cambodia had one of the highest TB prevalence rates in the world [16]. Given that the TB burden remains high in Cambodia, there is a need for an updated assessment of the levels and trends in TB morbidity and mortality in the country.

This updated assessment provides the first opportunity to empirically investigate Cambodia’s progress towards global targets defined by the WHO End TB strategy that aims to end the TB epidemic by reducing TB mortality by 95% and incidence by 90% by 2035 [17, 18]. Interim milestones for 2020 have passed, which were defined as reductions of 20% of the incidence rate and 35% in the number of deaths. Further, previous TB burden enumeration exercises in Cambodia have yet to incorporate the contribution of potentially modifiable risk factors, such as tobacco smoke, alcohol consumption, and diabetes. This is particularly important for Cambodia as challenges in treating TB in the country are partly due to the high prevalence of risk factors such as smoking [19].

Thus far, Cambodia’s national TB program has increased directly observed treatment short course (DOTS) coverage, expanded active case finding (ACF), and defined explicit priorities in identifying cases among high-risk populations to manage TB burden [20–23]. The national program has also made progress in bridging treatment gaps by integrating HIV and TB services to screen for TB among persons living with HIV [24]. Cambodia’s TB strategic plan for 2021 through 2030 includes increasingly early identification of TB cases, providing sustained and equitable access to high quality TB services, and preventing TB transmission through awareness and behavioral communication strategies [25, 26]. The national TB program mainly concerns key and vulnerable populations including people living with HIV, elderly people (aged 55 and older), children, and people with diabetes [27, 28]. A systematic assessment of TB trends with the inclusion of these key populations may further assist in monitoring Cambodia’s national TB goals and priorities.

This study therefore aims to examine the levels and trends in TB burden in Cambodia from 1990 to 2019 by leveraging the Global Burden of Diseases, Injuries, and Risk Factors Study (GBD) 2019 study [2]. We focus on evaluating progress towards the 2020 WHO End TB interim milestones by providing estimates of TB incidence and mortality by HIV status, age, and sex. Finally, we estimate the number of TB deaths, among individuals without HIV coinfection, attributable to the independent effects of risk factors, including smoking, alcohol consumption, and diabetes. Results from this analysis will provide robust and detailed evidence around the plausibility of eliminating TB in Cambodia, while helping to inform impactful policy and program decision-making.

## Methods

The GBD and tuberculosis (TB) burden estimation methods in the GBD have been published in detail [1–3, 29]. Input data sources for estimating the TB burden and risk factors for Cambodia specifically are available in the Additional file 1: Table S1.

Briefly, we used the Cause of Death Ensemble (CODEm) modeling framework to estimate deaths due to TB among individuals without HIV coinfection. CODEm is a Bayesian hierarchical ensemble modeling platform that leverages available cause-of-death data and information from 16 covariates (see Additional file 1: pp 11; Table S2) to derive mortality estimates for more than 200 countries including Cambodia from 1990 to 2019 [30]. TB deaths among individuals with HIV coinfection were established using a population attributable fraction approach (Additional file 1, pp 12–14).

Estimates of TB incidence and prevalence were generated using the DisMod-MR 2.1 (disease-model-Bayesian meta-regression) modeling tool [31]. DisMod-MR 2.1 is a Bayesian disease modeling tool that leverages all available morbidity and mortality data, the epidemiological relationships between disease parameters, and spatial relationships to output morbidity estimates. We distinguished morbidity estimates between TB and HIV coinfection and TB without HIV by applying the proportion of cases of TB and HIV coinfection among all cases of TB (Additional file 1, pp 25).

To analyze the attributable burden of TB among individuals without HIV coinfection due to risk factors, population attributable fractions (PAFs) were computed within the comparative risk assessments framework of the GBD [32] using the following: prevalence estimates for exposure to risk factors (smoking, alcohol consumption, and diabetes), the relative risk of TB mortality as an outcome of exposure to each risk factor, and the theoretical minimum risk exposure level. TB mortality attributable to each risk factor was computed by multiplying the PAF by the number of TB deaths for each risk–outcome pair for a given age, sex, and year.

### Data presentation

We computed age-standardized rates for incidence, mortality, and PAFs using the GBD world population age standard [33]. For changes over time, we present annualized rates of change as the difference in the natural log of the values at the start and end of the time interval divided by the number of years in the interval. We provide annualized rates of change for the following time intervals: 1990–2005, 2005–2015, and 2015–2019. Percent changes in all-form TB deaths and age-standardized all-form TB incidence rates from 2015 to 2019 were also presented to evaluate progress toward the 2020 WHO End TB interim milestones. We provide 95% uncertainty intervals (UIs) for every estimate based on the 2.5th and 97.5th percentiles of the posterior distributions carried over from each step in the modeling strategy.

## Results

### Tuberculosis burden in Cambodia

**Fig. 1:**
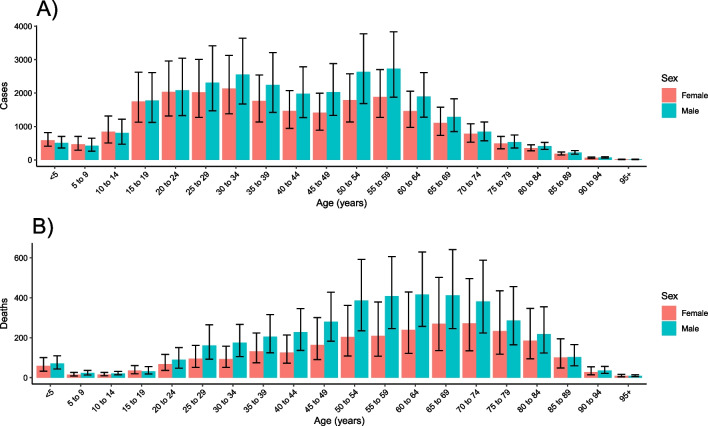
Age-sex distribution of all-form tuberculosis incident cases **A** and deaths **B** in Cambodia, 2019

In 2019, we estimated 6500 (95% UI 4830–8680) deaths due to all-form TB (Table 1), 50.0 (43.8–57.8) thousand all-form TB incident cases (Table 2), and 81.1 (70.5–93.4) thousand prevalent cases of active TB in Cambodia. Of these all-form TB deaths and incident cases in 2019, 457 (295–649) deaths and 2890 (2530–3340) incident cases were due to HIV-TB (Additional file 1: Table S5). Among individuals without HIV coinfection, we estimated 6050 (4410–8100) deaths due to TB and 47.1 (41.3–54.5) thousand TB incident cases (Additional file 1: Table S5). Approximately, 61.1% (46.2–71.1) of all-form deaths and 54.7% (53.0–56.5) of all-form incident cases occurred among males in 2019. Most all-form TB incident cases (79.9% [95% UI 75.8–83.4]) and deaths (57.6% [52.3–62.8]) in 2019 were in individuals aged 15–64 years (Fig. 1).

**Table 1:** All-form tuberculosis deaths and age-standardized rates of all-form tuberculosis mortality per 100,000 population in Cambodia by sex, 1990–2019

	Male		Female		Both	
Year	Deaths	Mortality rate (per 100,000)	Deaths	Mortality rate (per 100,000)	Deaths	Mortality rate (per 100,000)
1990	5170 (3800–6140)	217.1 (160.9–258.3)	4230 (1930–6210)	125.7 (58.2–184.6)	9400 (6860–11,800)	164.1 (119.5–204)
1991	5220 (3860–6140)	214.8 (159.4–253.9)	4280 (2000–6250)	124.5 (58.3–181.2)	9500 (7000–11,900)	162.3 (120.2–201.6)
1992	5310 (3950–6200)	213.6 (156.5–250.7)	4340 (2100–6270)	123.6 (59.1–178.6)	9650 (7170–12,000)	161.2 (120.6–200)
1993	5470 (4050–6360)	214.3 (157.9–250.3)	4460 (2250–6420)	124.1 (61.7–178.8)	9930 (7420–12,200)	161.8 (121.3–198.9)
1994	5700 (4310–6650)	217.7 (161.7–255.7)	4630 (2430–6570)	125.7 (64–179.2)	10,300 (7820–12,700)	164.1 (123.3–201.9)
1995	5990 (4590–7000)	222.2 (166.3–261.7)	4800 (2630–6770)	127 (67–180.4)	10,800 (8230–13,200)	166.7 (127–205.3)
1996	6280 (4820–7360)	226 (168.1–267.7)	5000 (2840–6990)	129.1 (71.5–182)	11,300 (8690–13,800)	169.5 (129–208.7)
1997	6600 (5040–7750)	230.8 (171.7–272.3)	5190 (3060–7210)	130.6 (73.3–182.5)	11,800 (9070–14,400)	172.4 (131.4–211.5)
1998	6930 (5220–8210)	234.8 (174.5–279.2)	5390 (3240–7420)	131.9 (76.9–184.5)	12,300 (9440–15,100)	174.8 (133–213.8)
1999	7140 (5330–8500)	235.2 (174–279.8)	5520 (3400–7620)	131.7 (78.3–183.6)	12,700 (9600–15,500)	174.8 (130.2–214.9)
2000	7290 (5380–8760)	233.6 (170.7–280.3)	5590 (3410–7710)	130.3 (77.7–182.1)	12,900 (9640–15,800)	173.3 (127.4–214.6)
2001	7250 (5330–8780)	225.8 (163.5–273.2)	5550 (3420–7680)	125.6 (75.5–177.5)	12,800 (9550–15,800)	167.3 (123.4–207.4)
2002	7070 (5170–8600)	213.9 (152.9–260.3)	5390 (3350–7520)	118.8 (71.3–170.9)	12,500 (9250–15,600)	158.3 (116.5–199)
2003	6800 (4910–8350)	200.2 (140.4–246)	5150 (3200–7310)	110.6 (66.6–160.4)	11,900 (8750–15,200)	147.8 (107.5–186.5)
2004	6520 (4670–8080)	187 (131.1–233.7)	4880 (2980–7070)	102.3 (61.5–151.2)	11,400 (8320–14,600)	137.4 (99.5–174.9)
2005	6220 (4420–7800)	174.2 (120.8–219)	4590 (2780–6790)	94 (56–141.7)	10,800 (7860–13,800)	127.2 (91.8–163.2)
2006	5920 (4190–7440)	162.2 (111.8–204.2)	4300 (2640–6410)	86.3 (52.3–130.2)	10,200 (7490–13,100)	117.7 (85.6–151.4)
2007	5710 (4030–7190)	152.7 (104.8–191.5)	4050 (2520–6020)	79.4 (48.8–120)	9760 (7170–12,500)	109.8 (80.9–140)
2008	5550 (3900–6950)	144.7 (99–182)	3820 (2410–5660)	73.3 (45.7–110.7)	9370 (6950–11,900)	102.8 (76.2–130.4)
2009	5430 (3840–6770)	138.3 (95–172.8)	3640 (2330–5360)	68.3 (43.4–103.6)	9070 (6820–11,500)	97.3 (72.7–122.9)
2010	5320 (3760–6620)	132.1 (92–164.7)	3510 (2270–5070)	64.3 (41.1–96.3)	8820 (6700–11,000)	92.3 (69.8–115.6)
2011	5140 (3680–6420)	124.9 (88.3–156.2)	3350 (2180–4880)	60.2 (38.8–89.7)	8490 (6470–10,700)	86.9 (66.2–109.1)
2012	4970 (3550–6220)	117.6 (83.3–146.7)	3220 (2100–4700)	56.4 (36.6–83.9)	8190 (6240–10,400)	81.6 (62.5–103.1)
2013	4790 (3460–6000)	110.4 (79.5–137.5)	3100 (2000–4540)	52.8 (34–78.9)	7890 (5970–10,100)	76.5 (58.4–96.8)
2014	4640 (3350–5830)	103.8 (75.1–130.3)	2990 (1920–4480)	49.7 (31.7–74.6)	7630 (5750–9840)	71.9 (54.6–91.8)
2015	4480 (3250–5710)	97.6 (71.1–123.2)	2890 (1860–4380)	46.7 (29.9–71.2)	7370 (5580–9440)	67.6 (51.8–86.6)
2016	4330 (3110–5540)	91.5 (66–116.1)	2790 (1790–4290)	43.9 (28.1–68.1)	7120 (5380–9260)	63.4 (48.1–81.7)
2017	4160 (2990–5320)	85.6 (61.7–108.6)	2690 (1710–4200)	41.2 (26.2–64.6)	6860 (5130–8970)	59.4 (44.9–76.6)
2018	4050 (2900–5260)	80.9 (58.4–103.9)	2620 (1660–4160)	39 (24.6–61.9)	6670 (4940–8770)	56.1 (41.9–72.6)
2019	3950 (2800–5160)	76.8 (54.6–99.2)	2560 (1610–4110)	37 (23.4–59.4)	6500 (4830–8680)	53.3 (39.9–69.4)

**Table 2:** All-form tuberculosis incident cases and age-standardized rates of all-form tuberculosis incidence per 100,000 population in Cambodia by sex, 1990–2019

	Male		Female		Both	
Year	Cases	Incidence rate (per 100,000)	Cases	Incidence rate (per 100,000)	Cases	Incidence Rate (per 100,000)
1990	19,400 (17,400–21,600)	600.6 (537.8–668.1)	23,800 (21,000–26,800)	537.3 (476.1–600.7)	43,200 (38,400–48,200)	560.7 (501.1–624.7)
1991	19,900 (17,900–22,100)	599.6 (537.7–666)	24,300 (21,600–27,300)	536.4 (477.3–596.9)	44,200 (39,500–49,200)	559.6 (500–621.5)
1992	20,500 (18,500–22,800)	601 (538–670.9)	24,900 (22,200–27,600)	536.8 (480.3–598.6)	45,400 (40,600–50,400)	560.3 (502.6–622.1)
1993	21,200 (19,000–23,600)	604.8 (541.8–675.6)	25,500 (22,800–28,300)	538.4 (483.7–599.4)	46,700 (41,900–51,900)	562.7 (505.2–625.9)
1994	22,000 (19,600–24,500)	611 (547.6–685.4)	26,200 (23,400–29,200)	541.2 (487.1–602.9)	48,200 (43,200–53,500)	566.9 (510.3–631.1)
1995	22,900 (20,400–25,600)	619.4 (553.3–699.8)	26,900 (23,900–30,100)	545.2 (490.6–609.7)	49,800 (44,600–55,300)	572.8 (516.4–640.6)
1996	24,000 (21,400–26,800)	634.2 (568–712.1)	27,900 (24,800–31,200)	552.8 (495.8–616.9)	51,900 (46,500–57,700)	583.4 (526.1–649.1)
1997	25,500 (22,800–28,500)	655.7 (587.7–733.9)	29,000 (25,800–32,500)	564.3 (504.8–629.3)	54,500 (48,800–60,700)	599.2 (539.5–666)
1998	27,100 (24,100–30,300)	678.1 (607.7–757.7)	30,300 (26,900–34,000)	576.5 (513.9–645.3)	57,400 (51,200–64,100)	615.8 (551.7–687.2)
1999	28,500 (25,400–31,900)	695.5 (622.5–779.9)	31,500 (27,900–35,500)	586.2 (521.1–658.4)	60,000 (53,400–67,400)	628.8 (562.3–702.4)
2000	29,600 (26,200–33,200)	702.2 (625.3–788)	32,400 (28,700–36,700)	590.2 (523.8–665.2)	62,000 (55,200–69,700)	634 (565.4–710)
2001	30,100 (26,800–33,900)	697 (619.5–779.3)	32,900 (29,300–37,100)	586.4 (521.5–659.5)	63,100 (56,100–70,600)	629.5 (560.2–705.3)
2002	30,400 (27,000–34,200)	684.5 (607.1–765.3)	33,100 (29,200–37,400)	576.4 (511.8–645.2)	63,500 (56,200–71,200)	618.4 (550.6–692.5)
2003	30,600 (27,000–34,500)	668.1 (593–748.5)	33,000 (29,100–37,200)	562 (498.9–630.1)	63,600 (56,100–71,600)	603.3 (535–677.4)
2004	30,600 (26,900–34,700)	651.1 (577.3–732.9)	32,800 (28,800–36,700)	545.3 (483.9–611.8)	63,400 (55,800–71,400)	586.6 (520.5–659.4)
2005	30,800 (27,000–35,100)	636.7 (562–721.5)	32,400 (28,500–36,500)	528.4 (466.2–595)	63,200 (55,500–71,500)	571 (504.3–642.1)
2006	30,900 (27,100–35,300)	621.3 (547–704.6)	31,700 (27,900–35,700)	506.1 (447.7–570.5)	62,600 (54,900–70,900)	552 (488.8–620.5)
2007	30,600 (26,800–34,900)	601.1 (530.5–680.4)	30,400 (26,800–34,300)	475.8 (421.2–536.9)	61,000 (53,600–69,000)	526.5 (466.7–590.8)
2008	30,100 (26,400–34,100)	578.8 (511.8–654.1)	28,800 (25,300–32,600)	442.6 (392.1–499.5)	58,900 (51,900–66,700)	498.5 (441.2–560.1)
2009	29,700 (26,000–33,700)	557 (491.5–630.3)	27,400 (23,900–31,000)	412.1 (363.5–465.4)	57,000 (50,100–64,500)	472.2 (417.4–532.1)
2010	29,400 (25,800–33,500)	538.6 (474.2–610.6)	26,400 (23,000–30,100)	389.5 (343.1–441)	55,800 (48,900–63,300)	451.7 (399–509.8)
2011	29,200 (25,800–33,300)	521.6 (461.7–590.6)	25,900 (22,500–29,500)	372.7 (328.9–419.8)	55,100 (48,200–62,600)	435 (386.2–491.1)
2012	28,900 (25,400–32,900)	503.1 (446.3–570.8)	25,200 (22,000–28,700)	356.2 (315–402.3)	54,200 (47,700–61,500)	417.8 (371.3–473.4)
2013	28,600 (25,100–32,700)	484.1 (429.5–550.9)	24,700 (21,600–28,100)	340.7 (300.7–387.2)	53,200 (46,700–60,800)	401.1 (355.8–455.3)
2014	28,200 (24,700–32,600)	465.8 (411.1–532)	24,100 (21,000–27,700)	326.7 (288.2–373.2)	52,400 (45,900–60,100)	385.4 (341.6–438.7)
2015	28,000 (24,300–32,500)	449.1 (393.6–515)	23,800 (20,500–27,400)	314.9 (274.5–359.6)	51,700 (45,100–59,900)	371.7 (327.6–424.4)
2016	27,600 (24,200–31,700)	430.5 (380–488.9)	23,300 (20,300–26,600)	301.6 (265.3–343.3)	50,800 (44,400–58,300)	356.3 (315.6–403.5)
2017	27,400 (23,800–31,500)	416.3 (366.3–473.7)	22,900 (19,900–26,400)	291.5 (255.3–333.5)	50,300 (43,900–57,500)	344.6 (303.3–391.3)
2018	27,500 (23,900–31,600)	408.4 (359.4–464.2)	22,900 (19,900–26,100)	285.6 (251.2–325.2)	50,300 (44,000–57,900)	337.9 (298–384.1)
2019	27,400 (23,800–31,800)	399.9 (351.4–455.1)	22,600 (19,400–26,100)	278.7 (241.2–321.3)	50,000 (43,800–57,800)	330.5 (289–378.6)

**Fig. 2:**
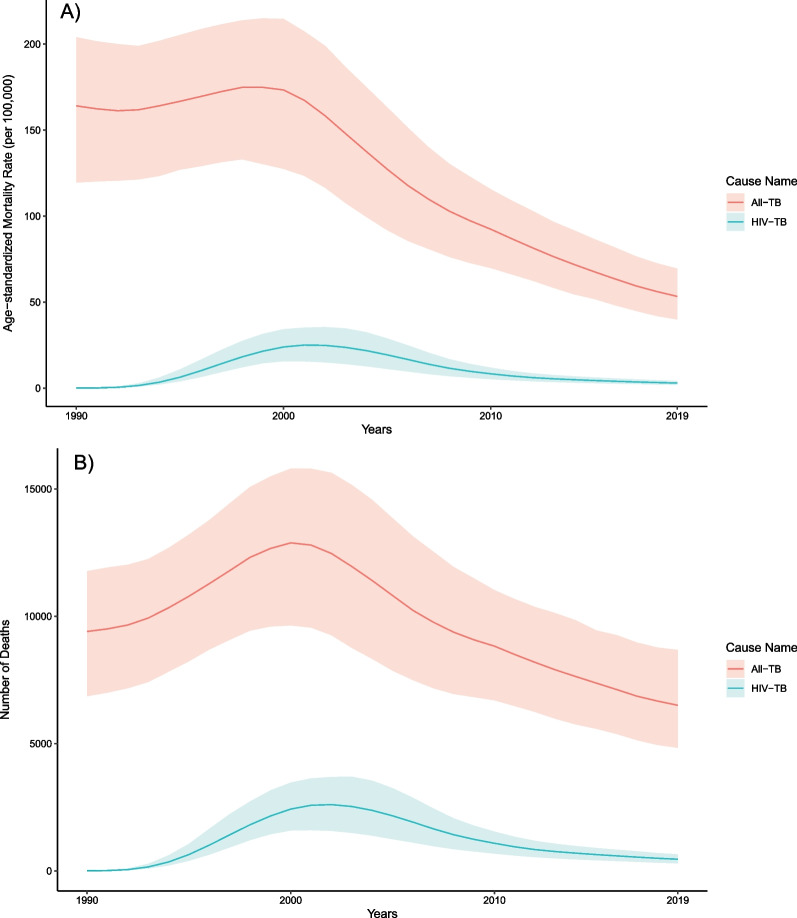
Temporal trends of age-standardized tuberculosis mortality rate per 100,000 population **A** and deaths **B** in Cambodia by HIV status, 1990–2019

**Fig. 3:**
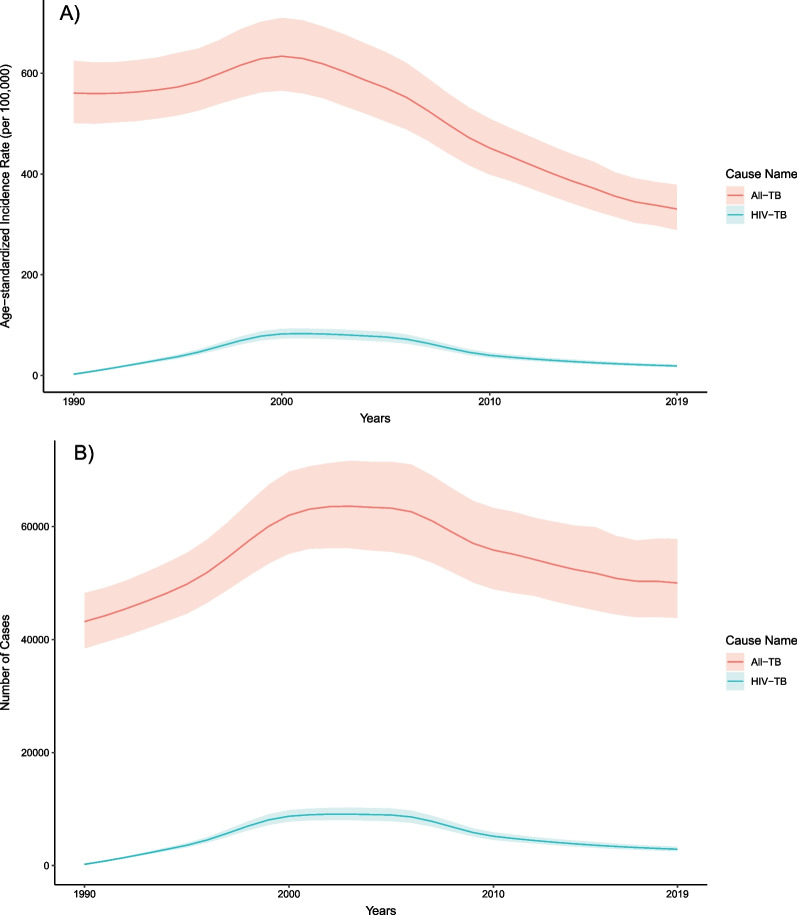
Temporal trends of age-standardized tuberculosis incidence rate per 100,000 population **A** and incident cases **B** in Cambodia by HIV status, 1990–2019

The age-standardized rates of all-form TB mortality, incidence, and prevalence per 100,000 population in 2019 were the following: 53.3 (39.9–69.4), 330.5 (289.0–378.6), and 549.6 (482.0–630.4). In 1990, these figures were 164.1 (119.5–204.0), 560.7 (501.1–624.7), and 799.7 (713.7–893.2) per 100,000 population, respectively (Figs. 2, 3). The age-standardized all-form TB mortality rate per 100,000 population in 2019 was 76.8 (54.6–99.2) among males and 37.0 (23.4–59.4) among females, and the age-standardized all-form TB incidence rate per 100,000 population was 399.9 (351.4–455.1) among males and 278.7 (241.2–321.3) among females (Tables 1, 2). The age-standardized male-to-female ratio for all-form TB mortality rate was 2.21 (1.13–3.37) and for all-form TB incidence rate was 1.44 (1.33–1.54).

### Changes in tuberculosis burden over time

In Cambodia, the annualized rate of change in age-standardized mortality of TB among individuals without HIV coinfection was -5.7% (95% UI − 8.4 to − 3.2) from 2015 to 2019, which was similar to the rate of change in 2005 to 2015 (− 5.4% [− 7.1 to − 3.6]) but faster than in 1990 to 2005 (− 2.8% [− 4.4 to − 1.2]). The rates of change for mortality were slightly quicker for the earlier time intervals for females (1990–2005: − 3.1 [− 5.0 to − 0.3], 2005–2015: − 5.9 [− 8.1 to − 3.2], 2015–2019: − 5.6 [− 8.8 to − 2.0]) than males (1990–2005: − 2.5 [− 4.3 to − 0.7], 2005–2015: − 4.9 [− 6.8 to − 3.1], 2015–2019: − 5.7 [− 8.9 to − 3.1]) (Table 3).

**Table 3:** Annualized rates of change for age-standardized rates of tuberculosis incidence and mortality among individuals without HIV coinfection in Cambodia

	Incidence			Mortality		
Years	Both	Female	Male	Both	Female	Male
1990–2005	− 0.8% (− 1.2% to − 0.4%)	− 0.9% (− 1.4% to − 0.5%)	− 0.6% (− 1.1% to − 0.2%)	− 2.8% (− 4.4% to − 1.2%)	− 3.1% (− 5.0% to − 0.3%)	− 2.5% (− 4.3% to − 0.7%)
2005–2015	− 3.6% (− 4.3% to − 2.8%)	− 4.7% (− 5.6% to − 3.8%)	− 2.5% (− 3.3% to − 1.7%)	− 5.4% (− 7.1% to − 3.6%)	− 5.9% (− 8.1% to − 3.2%)	− 4.9% (− 6.8% to − 3.1%)
2015–2019	− 2.6% (− 4.0% to − 1.3%)	− 2.8% (− 4.8% to − 0.9%)	− 2.6% (− 4.3% to − 0.8%)	− 5.7% (− 8.4% to − 3.2%)	− 5.6% (− 8.8% to − 2.0%)	− 5.7% (− 8.9% to − 3.1%)

The rates of change for mortality were larger than the reduction in the annualized rate of change in age-standardized TB incidence of − 2.6 (− 4.0 to − 1.3) from 2015 to 2019, which was slightly slower than the rate of change from 2005 to 2015 (− 3.6 [− 4.3 to − 2.8]) but significantly faster than in 1990 to 2005 (− 0.8 [− 1.2 to − 0.4]). Across all time intervals, the rates of change for incidence are quicker among females (1990–2005: − 0.9 [− 1.4 to − 0.5], 2005–2015: − 4.7 [− 5.6 to − 3.8], 2015–2019: − 2.8 [− 4.8 to − 0.9]) than males (1990–2005: − 0.6 [− 1.1 to − 0.2], 2005–2015: − 2.5 [− 3.3 to − 1.7], 2005–2019: − 2.6 [− 4.3 to − 0.8) (Table 3).

From 2015 to 2019, the number of all-form TB deaths in Cambodia decreased by − 11.8% (95% UI − 21.1 to − 2.3) from 7370 (5580–9440) deaths in 2015 to 6500 (4830–8680) (Tables 1, 4). Disaggregated by sex, the reduction was − 12.0% (− 22.2 to − 2.1) for males and − 11.6% (− 21.9 to − 1.6) for females in the same period. The age group with the largest decrease in all− form TB deaths between 2015 and 2019 was the under− 5 age group, with a reduction of − 37.5% (− 50.1 to − 22.5). The remaining under-15 age groups had reductions of over − 30%. Age groups from 15 to 49 years experienced nearly − 20% reductions, while remaining age groups above 50 years had reductions below − 10% for all-form TB deaths compared to 2015 (Table 4).

**Table 4:** Percent change in all-form tuberculosis incident cases and deaths and age-standardized rates of all-form tuberculosis incidence and mortality in Cambodia by sex and age from 2015 to 2019

	Incidence		Mortality	
	Percent change in incident cases	Percent change in incidence rate	Percent change in deaths	Percent change in mortality rate
Sex				
Both	− 3.3 (− 8.6–2.1)	− 11.1 (− 15.6–− 6.3)	− 11.8 (− 21.1–− 2.3)	− 21.2 (− 28.9–− 13)
Male	− 2.1 (− 8.9–5)	− 10.9 (− 16.5–− 4.6)	− 12 (− 22.2–− 2.1)	− 21.3 (− 30.5–− 12.9)
Female	− 4.7 (− 11.9–2.3)	− 11.5 (− 18.1–− 4.8)	− 11.6 (− 21.9–1.6)	− 20.7 (− 30–− 9)
Age Specific				
Under 5	− 19.1 (− 31.2–− 2.5)	− 19.6 (− 31.6–− 3.0)	− 37.5 (− 50.1–− 22.5)	− 37.9 (− 50.4–− 22.9)
5 to 9	− 18.7 (− 32.6–− 1.5)	− 22.3 (− 35.6–− 6)	− 34.1 (− 48.5–− 18.5)	− 37.1 (− 50.8–− 22.2)
10 to 14	− 17.6 (− 28.7–− 6)	− 20.1 (− 30.9–− 8.8)	− 30.4 (− 41.8–− 16)	− 32.5 (− 43.6–− 18.5)
15 to 19	− 20.4 (− 30.2–− 8.9)	− 17.2 (− 27.4–− 5.2)	− 25.3 (− 40.1–− 7)	− 22.2 (− 37.7–− 3.2)
20 to 24	− 15.7 (− 26–− 3.8)	− 12.8 (− 23.5–− 0.6)	− 21.6 (− 38.3–− 1.8)	− 18.9 (− 36.2–1.5)
25 to 29	− 6.4 (− 18.8–6.3)	− 10.6 (− 22.5–1.4)	− 20.7 (− 36.4–− 0.2)	− 24.3 (− 39.2–− 4.8)
30 to 34	0.9 (− 11.5–15)	− 10.9 (− 21.8–1.6)	− 19.2 (− 34.3–0.3)	− 28.6 (− 42–− 11.4)
35 to 39	9.4 (− 5.1–23.7)	− 10.8 (− 22.6–0.9)	− 14.9 (− 29.8–4.8)	− 30.5 (− 42.7–− 14.5)
40 to 44	3.2 (− 10.5–16.4)	− 8.6 (− 20.8–3.1)	− 18.1 (− 32.6–0.8)	− 27.5 (− 40.3–− 10.8)
45 to 49	− 7.6 (− 19.1–4.6)	− 8.3 (− 19.6–3.8)	− 20 (− 34.8–− 2.5)	− 20.6 (− 35.3–− 3.1)
50 to 54	− 0.1 (− 13.1–14.7)	− 8.4 (− 20.3–5.2)	− 11.9 (− 28.2–6.4)	− 19.2 (− 34.1–− 2.4)
55 to 59	5 (− 8.6–19)	− 9.7 (− 21.3–2.4)	− 5.7 (− 24.4–15.9)	− 18.9 (− 35–− 0.3)
60 to 64	11.3 (− 3–26.4)	− 8.7 (− 20.4–3.7)	− 0.8 (− 19.3–20.7)	− 18.6 (− 33.8–− 1)
65 to 69	8.3 (− 4.5–23.8)	− 8.7 (− 19.5–4.4)	− 5.9 (− 24–14.5)	− 20.7 (− 35.9–− 3.4)
70 to 74	10.4 (− 1.4–24)	− 10 (− 19.6–1.1)	− 2.1 (− 21.1–19.3)	− 20.2 (− 35.7–− 2.8)
75 to 79	0.9 (− 13–15.2)	− 9.3 (− 21.8–3.6)	− 9.7 (− 27.4–13.2)	− 18.8 (− 34.7–1.8)
80 to 84	2.1 (− 7.6–12.5)	− 9.7 (− 18.3–− 0.5)	− 8.1 (− 27.8–14.8)	− 18.8 (− 36.1–1.5)
85 to 89	3.9 (− 5.4–13.6)	− 9.6 (− 17.7–− 1.2)	− 6.4 (− 25.8–15.7)	− 18.5 (− 35.4–0.7)
90 to 94	4.4 (− 6.1–15.1)	− 9.7 (− 18.8–− 0.5)	1 (− 18–24.2)	− 12.7 (− 29.1–7.4)
95 plus	16 (− 0.2–30.8)	− 8.3 (− 21.1–3.4)	13.7 (− 8.9–40.7)	− 10.1 (− 28–11.3)

The age-standardized all-form TB incidence rate changed by − 11.1% (95% UI − 15.6 to − 6.3) from 371.7 (327.6–424.4) incident cases per 100,000 population in 2015 to 330.5 (289.0–378.6) per 100,000 population in 2019 (Tables 2, 4). The age-standardized all-form TB incidence rate changed by -10.9% (-16.5 to -4.55) among males and by -11.5% (-18.1 to -4.8) among females from 2015 to 2019. The under-25 age groups experienced the largest reductions in all-form TB incidence rate between 2015 and 2019 with the 5 to 9 age group having the sharpest decrease at 22.3% (6.0–35.6). The age groups 25 years and older experienced reductions near 10%, but many age groups did not have all-form TB incidence rates significantly lower than in 2015 (Table 4).

### Tuberculosis mortality attributable to individual risk factors

**Fig. 4:**
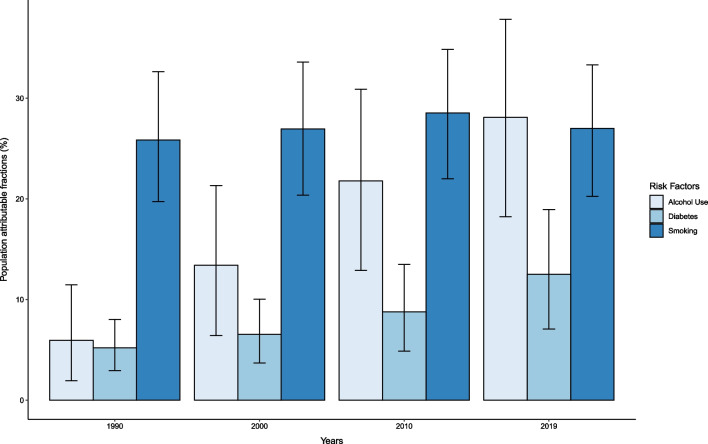
Age-standardized population attributable fractions of tuberculosis deaths due to alcohol use, smoking, and diabetes among individuals without HIV coinfection in Cambodia by year

In 2019, among individuals without HIV coinfection, the age-standardized population attributable fraction due to alcohol consumption was 28.1% (95% UI 18.2–37.9), due to smoking was 27.0% (20.2–33.3), and due to diabetes was 12.5% (7.1–19.0) for Cambodia (Fig. 4). The corresponding age-standardized population attributable fractions in 1990 were lower at 5.9% (1.9–11.5) for alcohol consumption, 25.8% (19.7–32.6) for smoking, and 5.2% (2.9–8.0) for diabetes (Fig. 4; Additional file 1: Table S6). In 2019, alcohol consumption accounted for 1790 (1120–2600) TB deaths, followed by smoking (1600 [1110–2210] TB deaths), and diabetes (699 [375–1100]) TB deaths) among individuals without HIV coinfection (Additional file 1: Table S6). Removing the combined effects of these risk factors would reduce all-form TB deaths to 3390 (2370–4772) in 2019 yielding a reduction of 54.2% (44.2 to 62.2) compared to 2015.

## Discussion

Tuberculosis (TB) burden in Cambodia remained large from a global perspective with an estimated age-standardized all-form TB mortality rate of 53.3 per 100,000 population (rank = 41/204 countries) and all-form TB incidence rate of 330.5 per 100,000 population in 2019 (rank = 19/204 countries). In the Southeast Asian region, Cambodia was among the top three countries with the highest TB incidence and mortality rates in 1990 and 2019. Despite considerable progress, Cambodia was slightly below the regional average for the percent reduction in TB incidence (51.7%) and TB mortality (68.6%) from 1990 to 2019 at 41.1% and 67.5%, respectively. Most TB deaths and cases occur among individuals without HIV coinfection in Cambodia. The reduction in TB mortality rate has been quicker than the reduction in TB incidence rate but the annualized rate of change has remained stable since 2005 for the TB mortality rate. The annual reduction for TB incidence rate had slowed down slightly, starting in 2015, compared to 2005.

Our results indicate that the annual reduction of age-standardized TB mortality in Cambodia has been improving since 1990, as the annual reduction was 5.7% from 2015 to 2019, 5.4% from 2005 to 2015, and 2.8% from 1990 to 2015. The near doubling of the annual rate of decline in the TB mortality rate between 2005 and 2015 may be primarily due to nationwide decentralization of directly observed treatment short course (DOTS) to health centers starting in 2004, resulting in near 100% DOTS coverage at the health center level [20], in doubling of case-finding [21], and in significant reductions in delays for access to TB services [34]. Another contributing factor to the observed progress may be the initiation and expansion of active case finding (ACF) programs in operational districts with high poverty rates and TB prevalence in Cambodia starting in 2005 [21]. These programs aim to identify missing TB cases and ensure early treatment initiation and have been shown to reduce TB burden [35]. Since 2005, the national TB program in Cambodia expanded ACF to high-risk populations and groups unlikely to be diagnosed when accessing health services [15, 22]. Moreover, Cambodia has piloted and implemented a myriad of interventions to improve and further expand ACF in the country during this timeframe [23, 36–38]. Early ACF programs have recently been strengthened to be more patient-driven due to widespread acceptance and success [39–42]. With the disruption of services due to the COVID-19 pandemic and subsequent reduction in TB case detection in many countries, strategies have been recommended to plan and monitor ACF activities during and beyond the pandemic [43]. Though early data suggests that the COVID-19 pandemic has resulted in 17,500 excess all-cause deaths in Cambodia [44], more data are needed to know the long-term impact of the COVID-19 pandemic on TB in Cambodia.

Despite significant progress in reducing TB mortality, Cambodia is not on track to achieve the End TB interim milestone for 2020 that aims to reduce the number of TB deaths by 35% in 2020 compared to 2015. We found that from 2015 to 2019, the number of all-form TB deaths decreased by 11.8%. While ACF interventions have improved access to TB services, other barriers remain high such as fear of high treatment costs [45], high rates of stigma toward those with TB [46], lack of awareness of TB-suggestive symptoms among people with TB [47], and indirect costs associated with long distances to health facilities [48], all contributing to significant delays in TB care. Widespread communication of TB awareness, of consciousness regarding TB stigma, and of the existing publicly available services for TB screening and treatment in Cambodia will be critical for meeting the End TB milestones.

Furthermore, we found that progress in decreasing TB incidence in Cambodia has been slower compared to the progress for TB mortality as we found that the annual reduction was 2.6% from 2015 to 2019, 3.6% from 2005 to 2015, and 0.8% from 1990 to 2005. Though the significant increase in the annual rate of change starting in 2005 indicates that interventions were beneficial in Cambodia, the slower progress for TB incidence compared to TB mortality suggests that there remain challenges for timely TB diagnoses. When diagnosis delays remain, untreated patients with TB can unknowingly transmit infections to healthy contacts [49]. Further, risk factors for progression from latent to active TB disease such as smoking are shown to be major challenges for TB control in Cambodia [19]. Studies have shown a lack of coordinated health service delivery for TB and TB risk factors [50, 51] in the country providing missed opportunities for early TB screening.

The slower progress in diminishing TB morbidity in Cambodia contributes to our finding that Cambodia is also not on track to achieve the End TB interim incidence milestone aiming to reduce TB incidence rate by 20% from 2015 to 2020. Our results indicate that the all-form TB incidence rate decreased by 11.1% from 2015 to 2019. To accelerate progress towards reducing TB transmission in Cambodia, decision-makers may consider using Xpert-MTB/RIF as evidence suggests the utilization of Xpert-MTB/RIF with ACF significantly increases case detection [52]. Previous work in Cambodia has shown that Xpert-MTB/RIF successfully complemented Cambodia’s ACF programs by increasing early TB diagnosis [53, 54] and was cost-effective [55, 56].

Finally, our results indicate that Cambodia’s national TB program could benefit from liaising with risk factor control initiatives to meet the milestones outlined by the WHO End TB Strategy. We found that the fraction of TB deaths attributable to alcohol and diabetes has increased significantly since 1990, while the fraction of TB deaths due to smoking remains unchanged. Further, we found that if the combined effects of smoking, alcohol consumption, and diabetes were removed, 54% of deaths would be reduced compared to 2015. Integrated services are therefore critical for reducing TB incidence as they could help prevent progression to active TB, increase early case detection, and prevent mortality. However, joint management of communicable diseases, like TB and TB risk factors, is limited in the local-level health system in Cambodia [57]. Potential integrated services may leverage Cambodia’s TB and HIV collaboration joint program as a framework for success, showing that over 90% of newly HIV positive individuals are referred to TB screening services [24]. Since risk factors such as diabetes, alcohol abuse, and smoking are significant predictors of progression from latent to active TB, this provides an opportunity for decision makers to combine their recent interest in bolstering latent TB infection diagnostic and treatment services among high-risk groups [24] by also targeting populations with these risk factors. Recognizing the synergy between smoking, alcohol, and diabetes with TB, integration of bi-directional screening strategies, support systems, and risk communication campaigns are urgently needed to reduce the burden of TB in Cambodia.

Despite different estimation strategies, the TB incidence estimates for Cambodia produced by GBD 2019 are similar to the WHO. GBD 2019 estimated 50.0 (95% UI 43.8–57.8) thousand all-form TB incident cases, while the WHO [4] estimated 47.0 (31.0–68.0) thousand incident cases for Cambodia in 2019. The similar estimates may partially be due to both groups using the available national TB prevalence survey data in Cambodia as inputs to their estimation methods. However, GBD 2019 and WHO differ in estimated TB mortality for Cambodia: GBD 2019 estimated 6500 (95% UI 4830–8680) deaths due to all-form TB and the WHO estimated 3300 (2200–4600) deaths in 2019. The difference in TB mortality estimates is due to different estimation strategies as the WHO utilized a case fatality ratio (CFR) approach using estimated incidence and CFR [58]. GBD 2019 utilized a myriad of predictive covariates (e.g., TB prevalence, latent TB infection prevalence, and TB strain prevalence-weighted transmission risk) and out-of-sample predictive validity assessment in the CODEm approach combined with statistical triangulation. The modeling strategy utilized in GBD 2019 yielded an implied CFR of approximately 13%, which was similar to neighboring countries including Indonesia (17%), Myanmar (14%), and Viet Nam (13%). The WHO generated a substantially lower implied CFR at 7%. Though the levels of TB incidence and mortality differ, GBD 2019 and the WHO have similar temporal trends as both groups estimated gradual declines in TB burden after the year 2000. Comparisons before the year 2000 cannot be examined since the WHO did not include the timeframe from 1990 to 1999 in their estimation period. The peak observed for Cambodia in the year 2000 in all-form TB incidence and mortality in our study is potentially a function of the HIV epidemic, as HIV burden was consistently at the highest levels during this timeframe in Cambodia based on modeling estimates from UNAIDS [59] and GBD 2019 [60].

The TB burden estimates in Cambodia should be interpreted in the context of the following limitations. First, the limitations of the underlying data may influence our results. For example, in the 2011 Cambodia TB prevalence survey, urban areas had lower participation rates than rural areas, and sputum samples were not collected from participants without chest radiography. Second, the availability of TB data in Cambodia is limited. To supplement the sparse TB data, we created covariates with evidence of a biological relationship or strong relationship with TB using data from various sources including population-based surveys such as Cambodia Demographic and Health surveys and data identified via systematic reviews. We also attempted to account for this limitation by using spatial relationships in our modeling, which leverages information from available data in surrounding countries. Our strategy further widened uncertainty intervals in years where data were sparse. Our estimates would improve if high-quality direct sources of TB burden measurement were consistently available in Cambodia. To base estimates more on direct measurement, Cambodia should improve surveillance systems by implementing legislation to make TB a nationally notifiable disease [24] and continue progress in creating a universal vital registration system [61].

Third, country-level results can mask subnational variation in the burden of TB and risk factors. Estimating more granular estimates, such as at the provincial level, may better inform resource allocation. Fourth, our risk factor analysis has not quantified TB burden attributable to other risk factors including malnutrition and indoor air pollution, mainly due to insufficient high-quality data. For example, TB burden attributable to malnutrition has not been characterized due to a lack of evidence for a causal link and limited data [62]. Similarly, there is limited availability of objectively measured longitudinal data to quantify TB burden attributable to indoor air pollution [63]. Lastly, our estimates represented the TB burden before the COVID-19 pandemic. The impact of the pandemic on TB transmission and mortality will require further investigation as data become available.

As Cambodia implements control initiatives from the 2021–2030 TB strategic plan, future research should monitor the impact of these policies on TB epidemiology and on progress towards the END TB targets. Future studies analyzing Cambodia’s TB policy goals could also benefit from examining trends in case detection, treatment access, and TB transmission. Further, there should be more data on the causal mechanisms for the slower annualized rate of change in incidence during the 2015–2019 period compared to the 2005–2015 period. One potential explanation is the rapid increase in the fraction of TB burden attributable to alcohol use and diabetes over time as identified in this study. However, further work is needed to examine the joint effect of risk factors and other factors such as alcohol regulation policies that might have contributed to the slower annualized rate of change.

## Conclusion

We leveraged the GBD framework to provide a comprehensive assessment of the levels and trends of TB burden in Cambodia from 1990 to 2019. Our findings illustrate that Cambodia is not on track to achieve the 2020 WHO End TB interim milestones despite substantial progress in reducing TB morbidity and mortality. Cambodia may benefit from supplementing ACF programs with widespread communication campaigns of TB awareness, consciousness regarding TB stigma, and of the existing publicly available financial services for TB treatment to increase early TB treatment initiation and prevent transmission. Furthermore, the Cambodia national TB program should examine the impact of risk factors, including alcohol consumption, smoking, and diabetes, and integrate with risk factor control initiatives of non- communicable disease (NCD) at local levels as a start to a TB and NCD initiative. Addressing these risk factors for TB may significantly accelerate progress toward achieving the WHO End TB targets and eliminating TB in Cambodia.

## Supplementary Information

**Additional file 1: Table S1.** Input data used for modeling the burden of tuberculosis in Cambodia. **Figure S1. **Flowchart. **Table S2.** Candidate covariates and priors evaluated in CODEm for tuberculosis. **Figure S2.** Flowchart. **Figure S3.** Flowchart. **Table S3.** Crosswalk adjustment factors for Tuberculosis prevalence surveys. **Figure S4.** Example of statistical triangulation and model fit to the data in DisMod MR 2.1 among males in Cambodia. **Table S4.** Beta coefficients and exponentiated values from the DisMod model. **Table S5.** Tuberculosis deaths and incident cases and age-standardized rates of tuberculosis mortality and incidence per 100,000 population by HIV status in Cambodia, 1990–2019. **Table S6.** Tuberculosis deaths attributable and age-standardized population attributable fractions to smoking, alcohol use, and diabetes among individuals without HIV coinfection in Cambodia, 1990–2019. **Table S7. **Tuberculosis age-standardized population attributable fractions to smoking, alcohol use, and diabetes among males and females without HIV coinfection in Cambodia, 1990–2019. **Figure S5.** Temporal trends of age-standardized tuberculosis mortality rate per 100,000 population **A** and deaths **B** in Cambodia by HIV status and sex, 1990–2019. **Figure S6.** Temporal trends of age-standardized tuberculosis incidence rate per 100,000 population **A** and incident cases **B** in Cambodia by HIV status and sex, 1990–2019. **Figure S7.** Age-standardized population attributable fractions of tuberculosis deaths due to alcohol use, smoking, and diabetes among individuals without HIV coinfection in Cambodia by year and sex.

## Data Availability

Data and code used in this analysis available at the Global Health Data Exchange GBD 2019 website: http://ghdx.healthdata.org/gbd-2019.

## Abbreviations

WHO: World Health Organization
TB: Tuberculosis
LMIC: Low-income and middle-income countries
DOT: Directly observed therapy
ACF: Active case finding
GBD: Global Burden of Diseases, Injuries, and Risk Factors Study
HIV: Human immunodeficiency virus
CODEm: Cause of Death Ensemble
DisMod-MR: Disease-model-Bayesian meta-regression
MI: Mortality-to-incidence
PAF: Population attributable fraction
UI: Uncertainty Interval
CFR: Case-fatality ratio
UNAIDS: United Nations Program on HIV/AIDS
NCD: Non-communicable disease
