# Supplementary material for: Progress towards the 2020 milestones of the end TB strategy in Cambodia: estimates of age and sex specific TB incidence and mortality from the Global Burden of Disease Study 2019
Source: BMC Infect Dis. 2022 Dec 3;22:904. doi: 10.1186/s12879-022-07891-5 (PMC9719136; doi:10.1186/s12879-022-07891-5)
Supplement: Supplementary file 1 — Additional file 1: Table S1. Input data used for modeling the burden of tuberculosis in Cambodia. Figure S1. Flowchart. Table S2. Candidate covariates and priors evaluated in CODEm for tuberculosis. Figure S2. Flowchart. Figure S3. Flowchart. Table S3. Crosswalk adjustment factors for Tuberculosis prevalence surveys. Figure S4. Example of statistical triangulation and model fit to the data in DisMod MR 2.1 among males in Cambodia. Table S4. Beta coefficients and exponentiated values from the DisMod model. Table S5. Tuberculosis deaths and incident cases and age-standardized rates of tuberculosis mortality and incidence per 100,000 population by HIV status in Cambodia, 1990–2019. Table S6. Tuberculosis deaths attributable and age-standardized population attributable fractions to smoking, alcohol use, and diabetes among individuals without HIV coinfection in Cambodia, 1990–2019. Table S7. Tuberculosis age-standardized population attributable fractions to smoking, alcohol use, and diabetes among males and females without HIV coinfection in Cambodia, 1990–2019. Figure S5. Temporal trends of age-standardized tuberculosis mortality rate per 100,000 population A and deaths B in Cambodia by HIV status and sex, 1990–2019. Figure S6. Temporal trends of age-standardized tuberculosis incidence rate per 100,000 population A and incident cases B in Cambodia by HIV status and sex, 1990–2019. Figure S7. Age-standardized population attributable fractions of tuberculosis deaths due to alcohol use, smoking, and diabetes among individuals without HIV coinfection in Cambodia by year and sex. [file 12879_2022_7891_MOESM1_ESM.docx]

# Additional file 1 to “Progress towards the 2020 milestones of the End TB Strategy in Cambodia, 1990–2019: evidence from the Global Burden of Disease Study 2019”

This appendix provides further methodological detail and results for “Progress towards the 2020 milestones of the End TB Strategy in Cambodia, 1990-2019: evidence from the Global Burden of Disease Study 2019”

All the material in the paper itself is novel although it builds off previous GBD works.^1–5^ However, parts of the supplemental methods appendix include sections adapted from the GBD Capstones published in *The Lancet* last year.^1,2^ References are provided for reproduced sections.

Table of Contents

**Methods2**

**Overview** **2**

**Table S1. Input data used for modeling the burden of tuberculosis in Cambodia** **3**

**Fatal Tuberculosis** **6**

**Figure S1. Flowchart** **6**

**Modelling fatal TB 6**

**Table S2. Candidate covariates and priors evaluated in CODEm for tuberculosis** ……................**11**

**Estimating HIV-TB12**

**Figure S2. Flowchart** **12**

**Non-fatal Tuberculosis** **15**

**Figure S3. Flowchart** **16**

**Modelling TB incidence** **17**

**Modelling TB prevalence** **18**

**Table S3. Crosswalk adjustment factors for Tuberculosis prevalence surveys** ………………………..**19**

**Figure S4. Example of statistical triangulation and model fit to the data in DisMod MR 2.1 among males in Cambodia** ………………………………………………………………………………………………………**23**

**Table S4. Beta coefficients and exponentiated values from the DisMod model** …………………….**24**

**HIV-TB incidence and prevalence** ………………………………………………………………….…………………………**25**

**Results26**

**Table S5. Tuberculosis deaths and incident cases and age-standardized rates of tuberculosis mortality and incidence per 100,000 population by HIV status in Cambodia, 1990–2019**  **26**

**Table S6. Tuberculosis deaths attributable and age-standardized population attributable fractions to smoking, alcohol use, and diabetes among individuals without HIV coinfection in Cambodia, 1990–2019** **28**

**Table S7. Tuberculosis age-standardized population attributable fractions to smoking, alcohol use, and diabetes among males and females without HIV coinfection in Cambodia, 1990–201930**

**Figure S5. Temporal trends of age-standardized tuberculosis mortality rate per 100,000 population (A) and deaths (B) in Cambodia by HIV status and sex, 1990–2019**  **32**

**Figure S6. Temporal trends of age-standardized tuberculosis incidence rate per 100,000 population (A) and incident cases (B) in Cambodia by HIV status and sex, 1990–2019**  **33**

**Figure S7. Age-standardized population attributable fractions of tuberculosis deaths due to alcohol use, smoking, and diabetes among individuals without HIV coinfection in Cambodia by year and sex**  **34**

*Overview*

We analyzed vital registration data, verbal autopsy data, sample-based vital registration data, and mortality surveillance data, using the Cause of Death Ensemble model (CODEm) to estimate TB mortality among HIV-negative individuals. We estimated HIV-TB mortality using a population attributable fraction approach taking into account baseline risk. We also used a population attributable fraction approach to estimate the fraction of HIV-negative TB deaths attributable to MDR-TB, and the fraction of HIV-TB deaths attributable to MDR-TB, respectively, and used these fractions to split TB deaths and HIV-TB deaths by drug-resistance type.

For estimating non-fatal TB, we analyzed all available data sources, including annual case notifications, prevalence surveys, population-based tuberculin surveys, and estimated TB cause-specific mortality, to generate internally consistent estimates of incidence, prevalence, and mortality using DisMod-MR 2.1, a Bayesian meta-regression tool. To distinguish HIV-TB from all forms of TB, we applied the proportions of HIV-TB cases among all TB cases estimated from a mixed-effects regression to TB incident and prevalent cases. We then applied the estimated proportions of TB cases with MDR-TB and HIV-TB cases with MDR-TB to our predicted TB cases and HIV-TB cases, respectively, to generate MDR-TB cases by HIV status.

All data sources used for modeling tuberculosis burden in Cambodia are available in Table S1.

Table S1. Input data used for modeling the burden of tuberculosis in Cambodia

| **Cause/Risk Factor** | **Measure** | **Year** | **Source** |
| --- | --- | --- | --- |
| Tuberculosis and latent tuberculosis infection | Prevalence | 2002 | Japan International Cooperation Agency, National Center for Tuberculosis and Leprosy Control (CENAT) (Cambodia), Research Institute of Tuberculosis/Japan Anti-Tuberculosis Association (RIT/JATA). Cambodia National Tuberculosis Prevalence Survey 2002 |
| Latent tuberculosis infection | Prevalence | 2004 | Norval PY, Roustit C, San KK. From tuberculin to prevalence survey in Cambodia. Int J Tuberc Lung Dis. 2004; 8(3): 299-305 |
| Tuberculosis | Prevalence | 2010–2011 | Japan International Cooperation Agency, Ministry of Health (Cambodia), National Center for Tuberculosis and Leprosy Control (CENAT) (Cambodia), Research Institute of Tuberculosis/Japan Anti-Tuberculosis Association (RIT/JATA), World Health Organization (WHO). Cambodia National Tuberculosis Prevalence Survey 2010-2011 |
| HIV-TB | Mortality | 2005–2014 | World Health Organization (WHO). WHO Tuberculosis Case Notifications. Geneva, Switzerland: World Health Organization (WHO) |
| HIV-TB | Prevalence | 2005–2014 | World Health Organization (WHO). WHO Tuberculosis Case Notifications. Geneva, Switzerland: World Health Organization (WHO) |
| HIV | Mortality / Prevalence | 2005–2006 | Macro International, Inc, National Institute of Public Health (Cambodia), National Institute of Statistics (Cambodia). Cambodia Demographic and Health Survey 2005-2006. Fairfax, United States of America: ICF International |
| HIV | Mortality | 2003–2013 | De La Mata NL, Ly PS, Van Nguyen K, Merati TP, Pham TT, Lee MP, Choi JY, Ross J, Law MG, Ng OT. Loss to follow-up trends in HIV-positive patients receiving antiretroviral treatment in Asia from 2003 to 2013. J Acquir Immune Defic Syndr. 2017; 1–13 |
| HIV | Mortality | 2011 | Van Griensven J, Thai S. Predictors of immune recovery and the association with late mortality while on antiretroviral treatment in Cambodia. Trans R Soc Trop Med Hyg. 2011; 105(12): 694-703 |
| Smoking | Population attributable fraction | 1999 | National Institute of Statistics (Cambodia), United Nations Development Programme (UNDP), World Bank. Cambodia Socio-Economic Survey 1999. Phnom Penh, Cambodia: National Institute of Statistics (Cambodia) |
| Smoking | Population attributable fraction | 2000 | Macro International, Inc, Ministry of Health (Cambodia), National Institute of Statistics (Cambodia). Cambodia Demographic and Health Survey 2000. Fairfax, United States of America: ICF International |
| Smoking | Population attributable fraction | 2003 | Centers for Disease Control and Prevention (CDC) and World Health Organization (WHO). Cambodia Global Youth Tobacco Survey 2003. United States: Centers for Disease Control and Prevention (CDC), 2003 |
| Smoking | Population attributable fraction | 2004 | National Institute of Statistics (Cambodia). Cambodia Smoking Behavior Survey 2004 |
| Smoking | Population attributable fraction | 2004 | Institute for Social Research, University of Michigan. Cambodia Elderly Survey 2004. Ann Arbor, United States of America: Institute for Social Research, University of Michigan |
| Smoking | Population attributable fraction | 2003–2005 | National Institute of Statistics (Cambodia), Statistics Sweden. Cambodia Socio-Economic Survey 2003-2005. Phnom Penh, Cambodia: National Institute of Statistics (Cambodia) |
| Smoking | Population attributable fraction | 2005–2006 | Macro International, Inc, National Institute of Public Health (Cambodia), National Institute of Statistics (Cambodia). Cambodia Demographic and Health Survey 2005-2006. Fairfax, United States of America: ICF International |
| Smoking | Population attributable fraction | 2005–2006 | Gallup. Cambodia World Poll 2005-2006 |
| Smoking | Population attributable fraction | 2006–2007 | National Institute of Statistics (Cambodia), Statistics Sweden. Cambodia Socio-Economic Survey 2006-2007. Phnom Penh, Cambodia: National Institute of Statistics (Cambodia) |
| Smoking | Population attributable fraction | 2010 | Ministry of Health (Cambodia), University of Health Sciences (Cambodia), World Health Organization (WHO). Cambodia STEPS Noncommunicable Disease Risk Factors Survey 2010 |
| Smoking | Population attributable fraction | 2010 | Centers for Disease Control and Prevention (CDC), World Health Organization (WHO). Cambodia Global Youth Tobacco Survey 2010. Atlanta, United States of America: Centers for Disease Control and Prevention (CDC) |
| Smoking | Population attributable fraction | 2010–2011 | ICF Macro, Ministry of Health (Cambodia), National Institute of Statistics (Cambodia). Cambodia Demographic and Health Survey 2010-2011. Fairfax, United States of America: ICF International |
| Smoking | Population attributable fraction | 2011 | Gallup. Cambodia World Poll 2011 |
| Smoking | Population attributable fraction | 2012 | Gallup. Cambodia World Poll 2012 |
| Smoking | Population attributable fraction | 2013 | Centers for Disease Control and Prevention (CDC), Ministry of Health (Cambodia), World Health Organization (WHO). Cambodia Global School-Based Student Health Survey 2013. Atlanta, United States of America: Centers for Disease Control and Prevention (CDC) |
| Smoking | Population attributable fraction | 2014 | ICF International, Ministry of Health (Cambodia), National Institute of Statistics (Cambodia). Cambodia Demographic and Health Survey 2014. Fairfax, United States of America: ICF International, 2017 |
| Smoking | Population attributable fraction | 2016 | Centers for Disease Control and Prevention (CDC), Ministry of Health (Cambodia), World Health Organization (WHO). Cambodia Global Youth Tobacco Survey 2016. Atlanta, United States of America: Centers for Disease Control and Prevention (CDC) |
| Smoking | Population attributable fraction | 2019 | World Health Organization (WHO). WHO Report on the Global Tobacco Epidemic 2019. Geneva, Switzerland: World Health Organization (WHO), 2019 |
| Diabetes | Population attributable fraction | 2010 | Ministry of Health (Cambodia), University of Health Sciences (Cambodia), World Health Organization (WHO). Cambodia STEPS Noncommunicable Disease Risk Factors Survey 2010 |
| Alcohol-Use | Population attributable fraction | 1960–1979 | World Health Organization (WHO). WHO Global Health Observatory - Recorded Alcohol Per Capita Consumption 1960-1979. Geneva, Switzerland: World Health Organization (WHO) |
| Alcohol-Use | Population attributable fraction | 1980–1999 | World Health Organization (WHO). WHO Global Health Observatory - Recorded Alcohol Per Capita Consumption 1980-1999. Geneva, Switzerland: World Health Organization (WHO) |
| Alcohol-Use | Population attributable fraction | 2000–2009 | World Health Organization (WHO). WHO Global Health Observatory - Recorded Alcohol Per Capita Consumption 2000-2009 by country. Geneva, Switzerland: World Health Organization (WHO) |
| Alcohol-Use | Population attributable fraction | 2015 | Ministry of Women's Affairs (Cambodia), National Institute of Statistics (Cambodia), World Health Organization (WHO). Cambodia WHO Multi-country Study on Women's Health and Domestic Violence Against Women 2015. Phnom Penh, Cambodia: National Institute of Statistics (Cambodia) |
| Alcohol-Use | Population attributable fraction | 2013 | Centers for Disease Control and Prevention (CDC), Ministry of Health (Cambodia), World Health Organization (WHO). Cambodia Global School-Based Student Health Survey 2013. Atlanta, United States of America: Centers for Disease Control and Prevention (CDC) |
| Alcohol-Use | Population attributable fraction |  | Euromonitor International. Euromonitor Passport - Alcoholic Drinks Statistics . London, United Kingdom: Euromonitor International |
| Tuberculosis | Incidence/Mortality |  | Input data sources for estimating covariates:  TB prevalence: <https://ghdx.healthdata.org/gbd-2019/data-input-sources?components=1&covariates=1165&locations=10>  Latent TB infection prevalence: <https://ghdx.healthdata.org/gbd-2019/data-input-sources?components=1&covariates=1148&locations=10>  TB strain prevalence-weighted transmission risk: <https://ghdx.healthdata.org/gbd-2019/data-input-sources?components=1&covariates=1222&locations=1>  Smoking prevalence: <https://ghdx.healthdata.org/gbd-2019/data-input-sources?components=1&covariates=145&locations=10>  Cigarettes per capita: <https://ghdx.healthdata.org/gbd-2019/data-input-sources?components=1&covariates=14&locations=10>  Litres of alcohol consumed per capita: <https://ghdx.healthdata.org/gbd-2019/data-input-sources?components=1&covariates=2297&locations=10>  Fasting plasma glucose: <https://ghdx.healthdata.org/gbd-2019/data-input-sources?components=1&covariates=28&locations=10>  Adult underweight proportion: <https://ghdx.healthdata.org/gbd-2019/data-input-sources?components=1&covariates=1100&locations=10>  Indoor air pollution: <https://ghdx.healthdata.org/gbd-2019/data-input-sources?components=1&covariates=105&locations=10>  Lagged distributed income: <https://ghdx.healthdata.org/gbd-2019/data-input-sources?components=1&covariates=57&locations=10>  Education (years per capita): <https://ghdx.healthdata.org/gbd-2019/data-input-sources?components=1&covariates=33&locations=10> |

*Fatal Tuberculosis^1^*

Figure S1^1^. Tuberculosis (TB) mortality: input data, analytical processes, and outputs


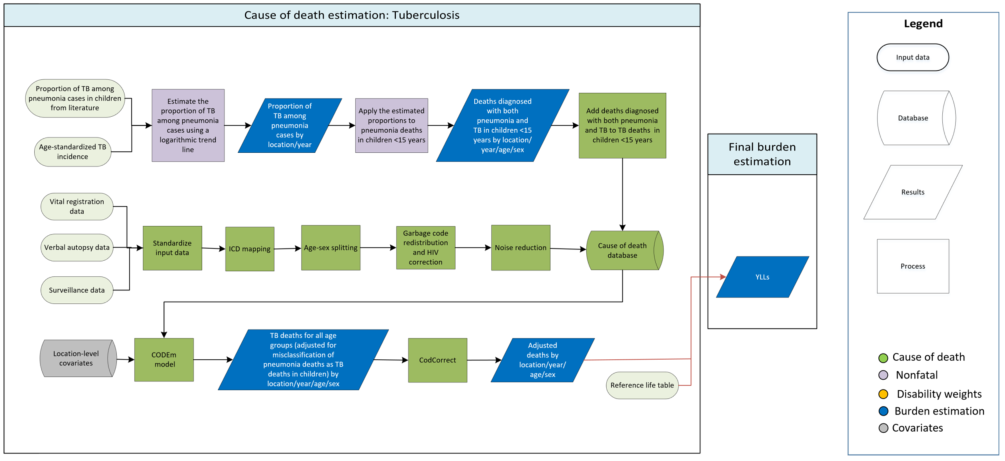


Note: Flowchart with code available at http://ghdx.healthdata.org/gbd-2019/code/cod-10

*Modelling fatal TB*

Input data for modelling tuberculosis (TB) mortality among HIV-negative individuals include vital registration, verbal autopsy, and surveillance data. The quality and comparability of the cause of death data were assessed and enhanced through multiple steps, including redistribution of garbage codes to underlying causes of death following GBD algorithms and adjustment for misclassified HIV deaths (ie, HIV deaths being assigned to other underlying causes of death such as TB because of stigma or misdiagnosis).^1^ Verbal autopsy data in countries with age-standardized HIV prevalence greater than 5% were removed because of a high probability of misclassification, as verbal autopsy studies have poor validity in distinguishing HIV deaths from HIV-TB deaths. GBD 2019 also assessed the overall cause of death (CoD) data quality for each country (based on completeness, garbage coding, cause list detail, and time periods covered), and assigned a quality score ranging from 0 stars (poorest) to 5 stars (best); a quality score of 4 to 5 is considered high quality.^1^

*Redistribution^1^*

A crucial aspect of enhancing the comparability of data for CoD is to deal with uninformative, so‐called garbage codes. Garbage codes to which deaths were assigned should not be considered as the underlying CoD--for example: “heart failure”, “ill‐defined cancer site”, “senility”, “ill‐defined external causes of injuries”, and “septicaemia”. The methods for redistributing these garbage‐coded deaths were outlined in detail in Naghavi et al^6^, and the underlying algorithm for redistributing deaths assigned to these codes has not changed since GBD 2013.

*Redistribution of TB CoD data: Regress garbage codes versus non-garbage codes^1^*

For each redistribution package, we defined the “universe” of data as all deaths coded to either the package’s garbage codes or the package’s redistribution targets for each country, year, age, and sex. We then ran a regression based on the following equation separately for each target group and sex:

$$TG_{crt}=\alpha+ \beta_{1}{Gar}_{crt}+\beta_{2}{Age}_{crt}{Gar}_{crt}+ \theta_{r}{Gar}_{crt}+\gamma_{r}+\varepsilon_{ct}$$

Where:

$TG_{crt}$= percentage of deaths within the given garbage code’s universe that were coded to a given target group, by country

${Gar}_{crt}$ = percentage of deaths within the given garbage code’s universe that were coded to a given set of garbage codes

${Age}_{CRT}=$age interaction term for the fixed effect on the interaction of garbage and age

$\alpha$ = constant

$\beta_{2}=$ slope coefficient describing the association between the interaction ${Age}_{crt}{Gar}_{crt}$ and $G_{crt}$

$\beta_{2}=$ slope coefficient describing the association between the interaction ${Age}_{crt}{Gar}_{crt}$ and $G_{crt}$

$\gamma_{r}=$ region-specific random intercept (or super-region if the random effect on region is not significant)

$\theta_{r}=$ region-specific random slope (or super-region if the random effect on region is not significant)

$\varepsilon_{ct}=$ standard error, normally distributed and calculated by bootstrapping

This regression was adjusted from GBD 2013 to include fixed effects on the interaction of garbage and age to ensure smooth age patterns. We made this decision after investigating diagnostic visualisations that showed unlikely gaps between proportions assigned to different age groups.

*Redistribution of TB CoD data: Computing redistribution uncertainty^1^*

We assigned redistribution variance to each data point by calculating residual variance from a regression predicting the percentage of garbage coded deaths redistributed to a cause, given the proportion of garbage codes we observed for that location, year, age, sex, cause, and the age standardized relative rate of major garbage codes across all causes. If there is a cause that has greater residual variance, we assume greater redistribution uncertainty.

To calculate variance, a dataset was generated containing percent garbage by location, year, age, sex, and cause, where percent garbage is determined by the equation:

$$pct_{garbage}=\frac{deaths_{redistributed}-deaths_{raw}}{deaths_{redistributed}}$$

A mixed-effect linear regression model was then fit to predict the logit percent of deaths from redistribution by age-standardized relative rate of major garbage codes.

$$logit\left( pct_{garbage_{ij}} \right)= \beta_{0}+\beta_{1}*\log\left( AS{R_{majorgarbage}}_{ij} \right)+\beta_{2}*15yearage_{ij}+\gamma_{1j}*\log\left( ASR_{majorgarbage_{ij}} \right)+u_{j}+e_{ij}, \theta_{\left\{ i \right\}}\sim N(0,\sigma^{2})$$

Where:

$i$ indexes dataset-location-year-age-sex-cause data points nested within $j$ groups by GBD region

${ASR}_{major garbage}$: age standardized relative rate of major garbage

Residual variance, as estimated by the MAD, was calculated for each cause, sex, and age. The next step was to use the residual variance to calculate uncertainty around each data point in the CoD database. First, we calculated the percent garbage of each data point by treating all deaths that could not be directly mapped to a GBD cause as garbage. Percent garbage was calculated as:

$$pct_{garbage}=\frac{deaths_{redistributed}-deaths_{corrected}}{deaths_{corrected}}$$

Where:

${deaths}_{corrected}$: deaths post $\text{misdiagnosis }$correction

${deaths}_{redistributed}$: deaths post redistribution

Residual variance was matched to each data point and 100 draws were sampled from a normal distribution by using the cause, age, sex, specific residual variance, and mean of 0. The logit transformed percent garbage was added to each value in the distribution. Each draw was then transformed out of logit space, and the post-redistribution deaths were calculated as

$$deaths=\frac{deaths_{corrected}}{1-pct\_garbage}$$

Draws of deaths were processed through noise reduction before calculating the final redistribution variance passed to modeling in CODEm, which was added to the total data variance. The mean of the draws was not used as the final estimate because it was found that the logit transformation biases the distribution of cause fractions higher than if only point estimates are used.

*Methods for correcting for a potential misclassification of tuberculosis deaths as pneumonia deaths in children*

First, we estimated the proportion of tuberculosis among pneumonia cases as a function of age-standardized TB incidence using data from eight clinical studies^7–14^ reporting the proportion of pneumonia cases that had tuberculosis (or the data to calculate them) and age-standardized TB incidence estimates. We used a logarithmic trend line to fit these data. In GBD 2019, we applied the estimated proportions to pneumonia deaths reported in data among children younger than 15 years to compute the number of deaths diagnosed with both pneumonia and TB, which were then added to child TB data. Following this correction in our input data, the CODEm model was ran to provide location-year-age-sex specific estimates.

*The Cause of Death Ensemble model (CODEm)*

TB mortality trends among HIV-negative individuals was modelled using the Cause of Death Ensemble modelling (CODEm) strategy, which is based on five general principles: identifying all available data, enhancing the comparability and quality of the dataset, developing a diverse set of possible models, assessing the predictive validity of all models, and selecting the models with the best performance in out‐of‐ sample predictive validity tests. Possible models were identified using a covariate selection algorithm that yielded many plausible combinations of covariates which were then run through four classes of models. These model classes include modeling natural log rates and logit cause fractions using mixed effects linear models and spatiotemporal Gaussian Process Regression models. This generated a large variety of models that competed in predictive validity tests. An ensemble of CODEm models that performed best on out-of-sample predictive validity tests was then selected. Details on how candidate models were developed, evaluated, and selection of best model are found elsewhere.^15^

Table S2: Candidate covariates and priors evaluated in CODEm for tuberculosis

|  | Covariate | Direction |
| --- | --- | --- |
| Level 1 | TB prevalence  Latent TB infection prevalence  TB summary exposure value (SEV) scalar  Liters of alcohol consumed per capita  Smoking prevalence  Cigarettes per capita  Fasting plasma glucose  TB strain prevalence-weighted transmission risk | +  +  +  +  +  +  +  + |
| Level 2 | Healthcare access and quality (HAQ) Index  Adult underweight proportion  Indoor air pollution  Outdoor air pollution  Population density | -  +  +  +  + |
| Level 3 | Log lag distributed income (LDI) per capita  Education (years per capita)  Socio-demographic Index (SDI) | -  -  - |

*Estimating fatal HIV-TB*

Figure S2^1^. HIV-Tuberculosis mortality: input data, analytical processes, and outputs


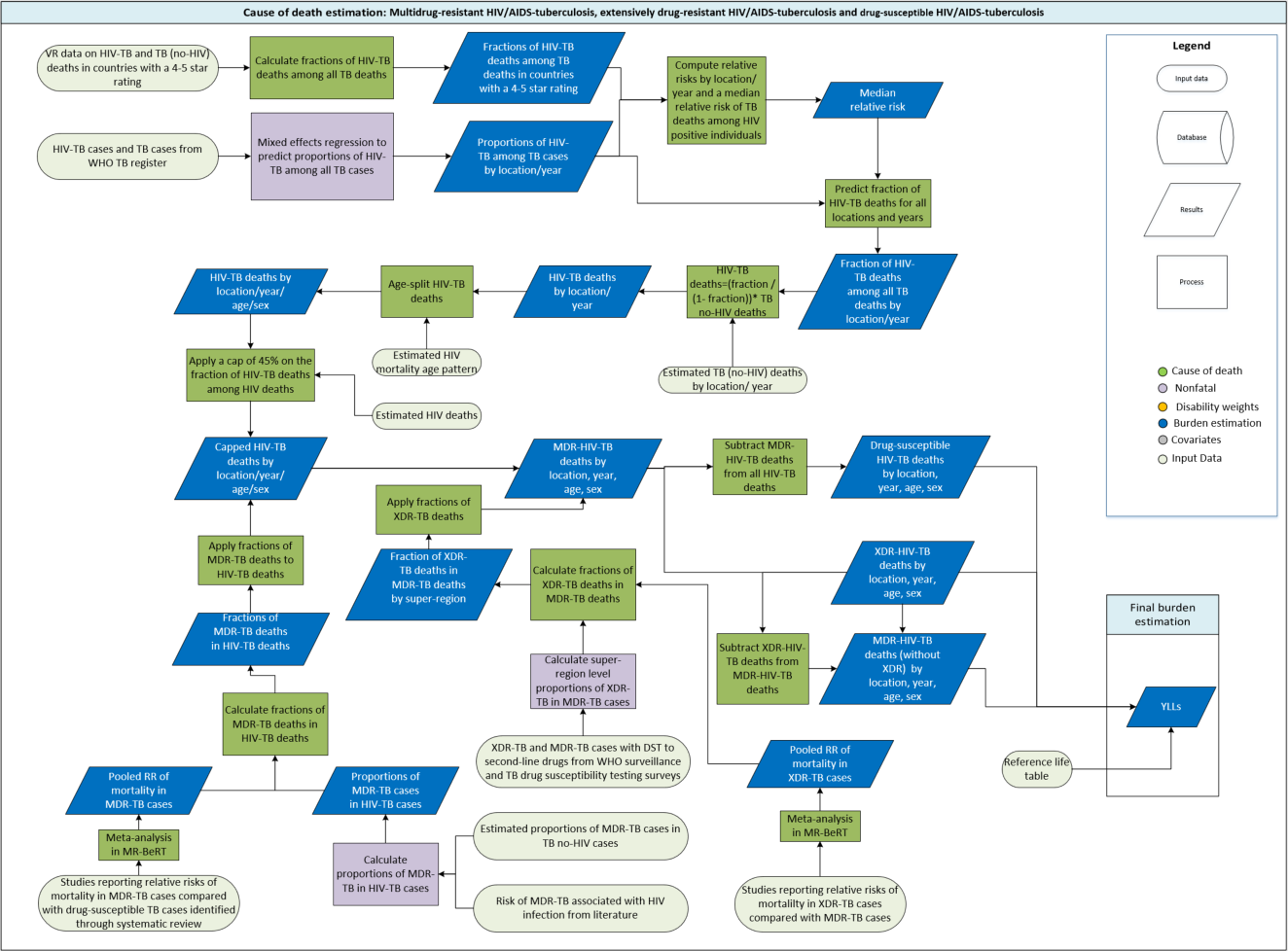


Note: Flowchart with code available at http://ghdx.healthdata.org/gbd-2019/code/cod-6

Input data include: (1) of vital registration data from countries with a four- or five-star rating where cause of death data for directly coded HIV-TB and tuberculosis (TB) were available, and (2) the number of TB cases (new and re-treatment) recorded as HIV-positive and the number of TB cases (new and re-treatment) with an HIV test result recorded in the TB register from the World Health Organization (WHO).

To determine TB deaths in HIV-positive individuals, we first computed the fraction of HIV-TB deaths among all TB deaths using vital registration data from countries with a four-or five-star rating. We also calculated the proportion of TB cases that are HIV-positive. We used these proportions as input data for a mixed effects regression to predict the proportions of HIV-TB cases among all TB cases for all locations and years using an adult HIV death rate covariate. We estimated the fraction of HIV-TB deaths among all TB deaths in each location and year ${(D}_{c,y})$, defined by:

$$D_{c,y}=\frac{P_{c,y}RR}{P_{c,y}RR+1-P_{c,y}}$$

where $P_{c,y}$ is the proportion of HIV-TB cases among all TB cases and *RR* is the relative risk of TB deaths in HIV positive individuals, defined by:

$$RR=\frac{D_{c,y}P_{c,y}-D_{c,y}}{D_{c,y}P_{c,y}-P_{c,y}}$$

We took the median relative risk (RR) from each calculation. We then applied the median RR and the predicted proportions of HIV-TB cases among all TB cases to get the fractions of HIV-TB deaths among all TB deaths for all locations and years. Location-year-specific HIV-TB deaths were then calculated using the following equation:

$${Deaths}_{HIV-TB}=\frac{D_{c,y}}{1-D_{c,y}}{Deaths}_{TB}$$

where ${Deaths}_{TB}$ is location-year specific deaths from the CODEm TB no-HIV model. Finally, we applied the age-sex pattern of the HIV mortality estimates to these HIV-TB deaths to generate location-year-age-sex-specific HIV-TB deaths. As the HIV-TB deaths were estimated based on the fraction of HIV-TB deaths among all TB deaths, the total number of HIV-TB deaths could exceed the total number of HIV deaths in some locations. To avoid this, we applied a cap of 45% on the fraction of HIV-TB deaths among HIV deaths, based on a review by Cox and colleagues, 2010^16^, and a systematic review and meta-analysis by Ford and colleagues, 2016.^17^

*Modelling non-fatal TB^1^*

Input data include annual case notifications, data from prevalence surveys, and estimated cause specific mortality (CSMR) of TB among HIV-positive and HIV-negative individuals. We divided the inputs on prevalence, incidence, and CSMR by the estimated latent TB infection (LTBI) prevalence weighted by the risk of progression to active TB in order to model TB among those at risk in each country. From these inputs, we modeled remission and used estimated remission to compute excess mortality to give more guidance to the model. We used DisMod-MR 2.1, the GBD Bayesian meta-regression tool that adjusts for differences in methods between data sources and imposes consistency between data for different parameters. We then multiplied the DisMod-MR 2.1 outputs by the prevalence of LTBI to get population-level estimates of incidence and prevalence. We explain in more detail below the preparation of each of the input data sources and the modelling in DisMod-MR 2.1. Updated systematic reviewers were done in GBD 2019 for TB prevalence surveys and LTBI tuberculin surveys. The search terms, number of studies identified, and number of studies included are shown in the table below:

Figure S3^1^. Tuberculosis (TB) morbidity: input data, analytical processes, and outputs


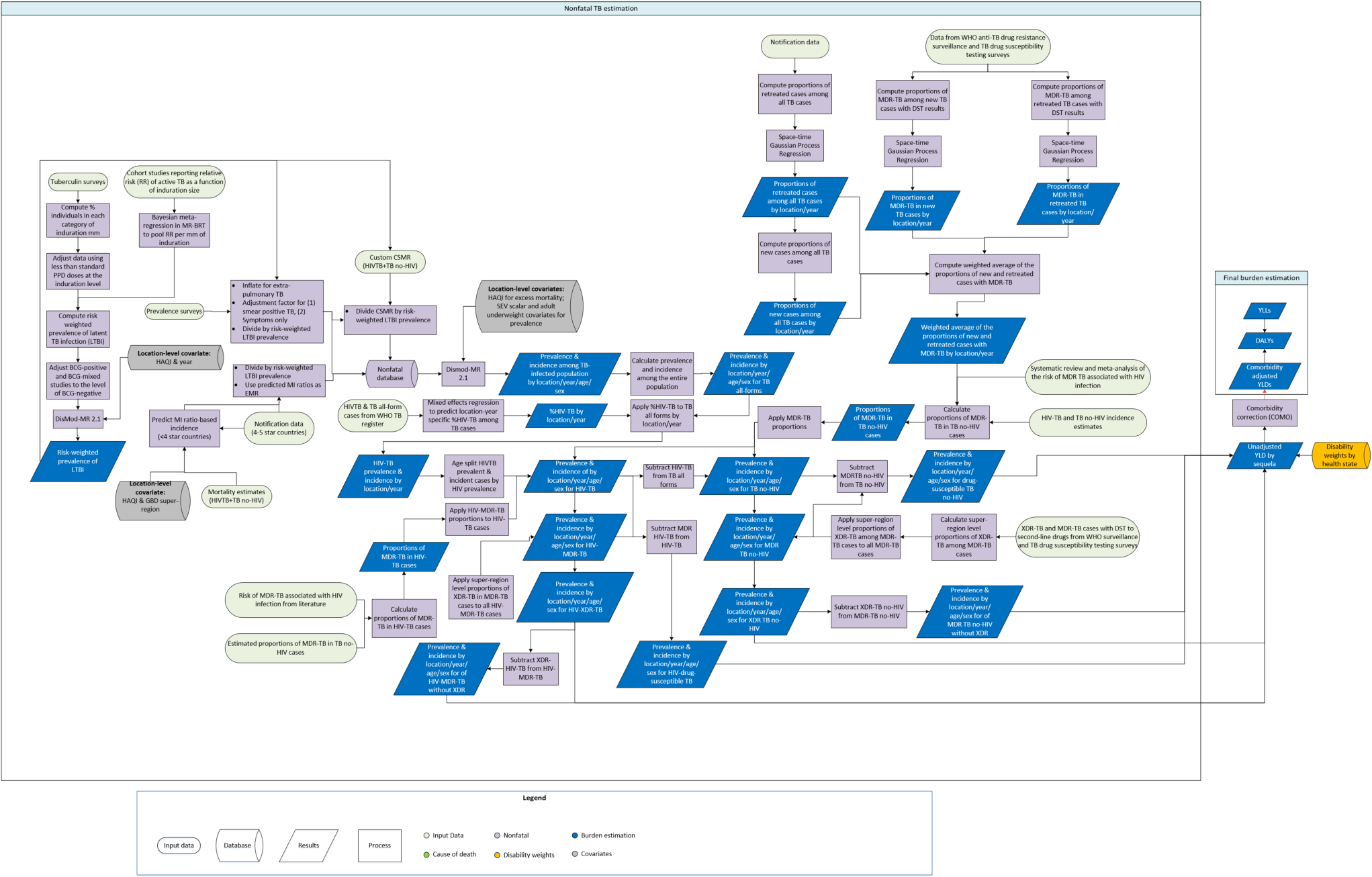
 Note: Flowchart with code available at http://ghdx.healthdata.org/gbd-2019/code/nonfatal-13

*Modelling TB incidence*

Incidence inputs were from two different sources: (1) incidence from notification data for countries with a four- or five-star rating on their cause of death data^6^ as a proxy for the quality of health-related administrative data systems, and (2) estimated incidence for countries with a less than four-star rating.

We used age- and sex-specific notifications (all new and relapse cases combined) in our analysis for countries with a 4 to 5-star rating. Starting from 2013, notified new cases and relapse cases are aggregated and reported together, and we used the data as they were reported. Prior to 2013, notification data were available by case type (new pulmonary smear-positive, new pulmonary smear-negative, and new extra-pulmonary) and there were missing age data, especially for younger age groups in some countries. We imputed the missing age groups for the three forms of TB notifications. Smear-positive age-specific notifications were inflated with the proportion smear-unknown and relapsed cases only reported at the country-year level. Some countries reported only pulmonary smear-positive cases for selected years. Missing smear-negative and extrapulmonary cases were predicted from the adjusted smear-positive cases using a seemingly unrelated regression. All three types of notifications were added together to represent TB-all-form incidence for countries with a four- or five-star rating.

To generate incidence estimates for locations with a less than four-star rating, we used the Meta-Regression with Bayesian Priors, Regularization, and Trimming (MR-BRT) model^18^ as the primary analytical engine to predict MI ratios. In the model, we used age and sex dummies and super-region fixed effects with MI ratios (logit transformed) from locations with a 4- or 5-star rating on causes of death as input data. The HAQ index^19^ was used as a covariate and anchoring the lower end of the HAQ index scale with a data point from a cohort study in the 1960s reporting that 49.2% of 126 untreated new pulmonary TB cases were dead at the end of the five-year follow-up period.^20^ Our model was then used to predict age-sex-specific MI ratios for all locations and years.

We then used the MI ratios and cause-specific mortality estimates to compute the incidence input for DisMod-MR 2.1 for locations with a less than four-star rating. Finally, we computed the age-sex-specific incidence of TB among the latent TB-infected population, using TB incidence as the numerator and our estimated risk-weighted latent TB infection prevalence as the denominator. Our final incidence estimate that is consistent with prevalence data and CSMR estimates was then generated using DisMod-MR 2.1.

*Modelling TB prevalence*

Data from prevalence surveys reporting on pulmonary smear-positive TB and bacteriologically positive TB were included. Because incidence data are for all forms of TB, we adjusted prevalence surveys to account for extrapulmonary cases. We ran a spatiotemporal Gaussian process regression to predict location-year-age-sex-specific proportions of extrapulmonary TB among all TB cases using data on the three forms of TB from the incidence data above. We then computed the extrapulmonary inflation factor as 1+( proportion of extrapulmonary TB /(1- proportion of extrapulmonary TB)), and applied it to data from prevalence surveys.

In GBD 2019, we used the MR-BRT model to derive adjustment factors for studies where the case definition was smear-positive TB rather than bacteriologically positive TB (reference). For the adjustment, we identified all prevalence surveys that provided comparisons of smear-positive TB and bacteriologically positive TB from the same sample. Overall, 16 prevalence surveys from Cambodia, China, Ethiopia, Gambia, India, Myanmar, South Korea, Philippines, Rwanda, and Vietnam were included as inputs in the MR-BRT model.^21–36^ The model also contained covariates for sex and age to reflect gradients across demographics. In GBD 2019 we also computed an adjustment factor to adjust studies that used symptoms only as a screening method compared to studies using both symptoms and chest X-ray during screening (reference). To derive the adjustment factor, we ran a MR-BRT model where data from six studies^34,37–41^ comparing prevalence between using symptoms only as opposed to symptoms and chest X-ray in the same population as input.

Finally, we computed the prevalence of TB among the TB-infected population, using TB prevalence as the numerator and our estimated risk-weighted LTBI prevalence as the denominator. We included two location-level covariates, namely, age-standardized adult underweight prevalence and log-transformed age-standardized Summary Exposure Variable (SEV) scalar for TB (a summary variable of the exposure levels of TB risk factors weighted by relative risk) to help inform variation of TB prevalence over year and geography.

Table S3. Crosswalk adjustment factors for Tuberculosis prevalence surveys

| Reference or alternative case definition | Gamma | Beta Coefficient,  Log (95% CI) | Adjustment factor* |
| --- | --- | --- | --- |
| Bacteriologically positive | 0.17 | --- | --- |
| Smear positive |  | -0.39 (-0.58 to -0.22) | 0.67 |
| Symptoms and chest X-ray | 0.01 | --- | --- |
| Symptoms only |  | -0.38 (-0.50 to -0.25) | 0.68 |

* *Adjustment factor is the transformed Beta coefficient in normal space, and can be interpreted as the factor by which the alternative case definition is adjusted to reflect what it would have been if measured as the reference.*

*Modelling TB remission and excess mortality*

In GBD 2019 we computed TB duration based on a systematic review of studies during the pre-chemotherapy era finding that duration from onset to cure or death is 3 years.^42^ To anchor the lowest end of TB duration we assumed a duration of 6 months based on treatment regimens. We then linearly interpolated between 6 months and 3 years across the HAQ index to compute TB duration for every country-year. The predicted duration values are shown in eTable 3. We converted duration into remission by taking the inverse (e.g. Remission = 1/duration). Using HAQ-based remission and estimated MI ratios, we computed excess mortality rate (EMR) with the following computation: EMR = MI*Remission (formula derived from Prevalence=Incidence*Duration).

*DisMod-MR 2.1*

*DisMod MR 2.1 description*

In GBD 2019, no substantial changes were made to DisMod-MR 2.1. The sequence of estimation occurs at five levels: global, super-region, region, country and, where applicable, subnational location. The super-region priors are generated at the global level with mixed-effects, nonlinear regression using all available data; the super-region fit, in turn, informs the region fit, and so on down the cascade. Subnational estimation was informed by the country fit and country covariates, plus an adjustment based on the average of the residuals between the subnational location’s available data and it’s prior. This mimicked the impact of a random effect on estimates between subnationals. At each level of the cascade, the DisMod-MR 2.1 enforces consistency between all parameters. Analysts have the choice to branch the cascade in terms of time and sex at different levels depending on data density. We used the default option to model TB, which is to branch by sex after the global fit but to retain all years of data until the lowest level in the cascade.

The coefficients for country covariates were re-estimated at each level of the cascade. For a given location, country coefficients were calculated using both data and prior information available for that location. In GBD 2019, we generated model fits for the years 1990, 1995, 2000, 2005, 2010, 2015, 2017, and 2019, and log-linearly interpolated estimates for the intervening years. The 95% uncertainty intervals were computed based on 1000 draws from the posterior distribution of the model using the 2.5th and 97.5th percentiles of the ordered 1000 values.

*DisMod-MR 2.1 likelihood estimation*

Analysts have the choice of using a Gaussian, log-Gaussian, Laplace or Log-Laplace likelihood function in DisMod-MR 2.1. We used the default log-Gaussian equation for the data likelihood, which is:

$$-log\left[ p\left( y_{j} | \Phi\right) \right]=\log\left( \sqrt{2\pi} \right)+\log\left( \delta_{j}+s_{j} \right)+\frac{1}{2}\left( \frac{\log\left( a_{j}+\eta_{j} \right)-\log\left( m_{j}+\eta_{j} \right)}{\delta_{j}+s_{j}} \right)^{2}$$

where, y_j_ is a ‘measurement value’ (i.e., data point); Φ denotes all model random variables; η_j_ is the offset value, eta, for a particular ‘integrand’ (prevalence, incidence, remission, excess mortality rate, cause-specific mortality rate) and a_j_ is the adjusted measurement for data point j, defined by:

$$a_{j}=e^{\left( -u_{j}-c_{j} \right)}y_{j}$$

where u_j_ is the total ‘area effect’ (i.e., the sum of the random effects at three levels of the cascade: super-region, region and country) and c_j_ is the total covariate effect (i.e., the mean combined fixed effects for sex, study level and country level covariates), defined by:

$$c_{j}=\sum_{k=0}^{K\left[ I\left( j \right) \right]-1} \beta_{I\left( j \right),k}\hat{X}_{k,j}$$

with standard deviation

$$s_{j}=\sum_{l=0}^{L\left[ I\left( j \right) \right]-1} \zeta_{I\left( j \right),l}\hat{Z}_{k,j}$$

where k denotes the mean value of each data point in relation to a covariate (also called x-covariate); I(j) denotes a data point for a particular integrand, j; β_I(j),k_ is the multiplier of the k^th^ x-covariate for the i^th^ integrand; $\hat{X}_{k,j}$ is the covariate value corresponding to the data point j for covariate k; l denotes the standard deviation of each data point in relation to a covariate (also called z-covariate); ζ_I(j),k_ is the multiplier of the l^th^ z-covariate for the i^th^ integrand; and δ_j_ is the standard deviation for adjusted measurement j, defined by:

$$\delta_{j}=log\left[ y_{j}+e^{(-u_{j}-c_{j})}\eta_{j}+c_{j} \right]-log\left[ y_{j}+e^{(-u_{j}-c_{j})}\eta_{j} \right]$$

Where m_j_ denotes the model for the j^th^ measurement, not counting effects or measurement noise and defined by:

$m_{j}=\frac{1}{B\left( j \right)-A(j)}\int_{A(j)}^{B(j)} I_{j}$(a) da

where A(j) is the lower bound of the age range for a data point; B(j) is the upper bound of the age range for a data point; and I_j_ denotes the function of age corresponding to the integrand for data point j.

*Internally consistent modelling in DisMod-MR 2.1*

For each location, we included the following as input in the DisMod model: case notifications for locations with a four- or five-star rating, predicted MI-ratio-based incidence for locations with a less than four-star rating, prevalence survey data where available, predicted excess mortality estimates, HAQ-based remission, and CSMR (TB and HIV-TB combined) by age and sex. DisMod then triangulated all these inputs to generate internally consistent estimates. Final results for locations may not be identical to the input data due to statistical triangulation in DisMod. An example of model fit to prevalence data in Cambodia is shown Figure S5. Beta coefficients and exponentiated values for covariates from the DisMod model are shown in eTable 3. The output from the DisMod model was for all forms of TB in TB-infected populations, including both HIV-negative and HIV-positive individuals. We computed the incidence and prevalence of TB among the entire population, by multiplying the prevalence of LTBI with the DisMod model estimates.

Figure S4. Example of statistical triangulation and model fit to the data in DisMod MR 2.1 among males in Cambodia.


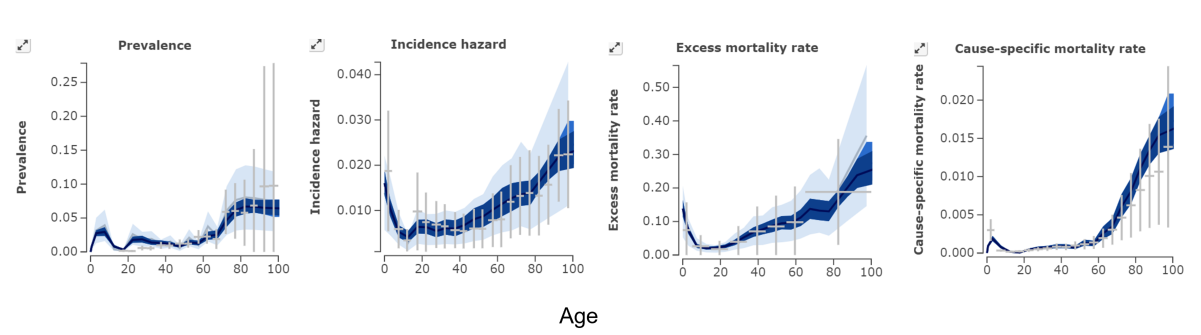


* The model was fit among the population living with tuberculosis infection. Prevalence was informed by data from the Cambodia national TB prevalence surveys. Cause-specific mortality was informed by covariates (Appendix Table S2) that leveraged various sources of population-based surveys and data. Incidence was informed by estimates of cause-specific mortality and mortality-to-incidence ratios. Excess mortality rates served as the link between morbidity and mortality data. The black line represents the final estimate with the dark blue shade representing the uncertainty interval around the final estimate. The lightly blue shade area represents the prior distribution.

Table S4. Beta coefficients and exponentiated values from the DisMod model

| Covariate | Parameter | Beta (95% CI) | Exponentiated beta (95% CI) |
| --- | --- | --- | --- |
| Sex (male) | Prevalence | 0.23 (0.19 to 0.26) | 1.26 (1.21 to 1.30) |
| Sex (male) | Incidence | 0.35 (0.35 to 0.35) | 1.42 (1.42 to 1.42) |
| Age-standardized proportion adult underweight | Prevalence | 2.08 (1.77 to 2.38) | 7.97 (5.90 to 10.86) |
| Age-standardized SEV scalar (log-transformed) | Prevalence | 0.75 (0.75 to 0.76) | 2.12 (2.12–2.14) |

*HIV-TB incidence and prevalence*

To distinguish HIV-TB from all forms of TB, we first estimated the proportions of HIV-TB cases among all TB cases using data on the number of TB cases recorded as HIV-positive and the number of TB cases with an HIV test result recorded in the WHO TB notifications register. We ran a mixed effects regression using the adult HIV death rate as a covariate to predict location-year-specific HIV-TB proportions, which were then applied to TB incident and prevalent cases from DisMod, to generate HIV-TB incident and prevalent cases by location and year. These cases were then age-sex split based on the age-sex pattern of estimated HIV prevalence by location-year to generate location-year-age-sex-specific HIV-TB incident and prevalent cases.

Table S5. Tuberculosis deaths and incident cases and age-standardized rates of tuberculosis mortality and incidence per 100,000 population by HIV status in Cambodia, 1990–2019

|  | **Male** | | | | **Female** | | | | **Both sexes** | | | |
| --- | --- | --- | --- | --- | --- | --- | --- | --- | --- | --- | --- | --- |
|  | **Deaths** | | **Incidence** | | **Deaths** | | **Incidence** | | **Deaths** | | **Incidence** | |
| **Year** | **TB** | **HIV-TB** | **TB** | **HIV-TB** | **TB** | **HIV-TB** | **TB** | **HIV-TB** | **TB** | **HIV-TB** | **TB** | **HIV-TB** |
| 1990 | 5160 (3800 - 6140) | 2 (1 - 6) | 19200 (17200 - 21400) | 170 (151 - 190) | 4230 (1930 - 6200) | 1 (1 - 2) | 23800 (20900 - 26800) | 51 (45 - 57) | 9400 (6860 - 11800) | 4 (2 - 8) | 42900 (38200 - 48000) | 221 (196 - 246) |
| 1991 | 5210 (3850 - 6140) | 8 (3 - 17) | 19300 (17400 - 21500) | 562 (505 - 623) | 4280 (2000 - 6250) | 4 (2 - 7) | 24100 (21400 - 27000) | 238 (215 - 263) | 9490 (6980 - 11900) | 11 (4 - 24) | 43400 (38800 - 48300) | 800 (720 - 886) |
| 1992 | 5280 (3910 - 6170) | 38 (23 - 67) | 19500 (17600 - 21700) | 984 (878 - 1090) | 4330 (2080 - 6260) | 12 (5 - 24) | 24400 (21800 - 27100) | 462 (413 - 511) | 9600 (7130 - 12000) | 50 (28 - 90) | 44000 (39300 - 48800) | 1450 (1290 - 1600) |
| 1993 | 5360 (3940 - 6260) | 102 (60 - 185) | 19800 (17800 - 22000) | 1420 (1280 - 1580) | 4410 (2210 - 6380) | 52 (30 - 87) | 24800 (22100 - 27500) | 716 (642 - 795) | 9770 (7250 - 12100) | 154 (91 - 277) | 44600 (40000 - 49500) | 2140 (1920 - 2370) |
| 1994 | 5480 (4080 - 6440) | 225 (136 - 404) | 20100 (17900 - 22400) | 1870 (1670 - 2070) | 4500 (2290 - 6470) | 124 (74 - 210) | 25200 (22500 - 28100) | 991 (888 - 1100) | 9980 (7490 - 12400) | 349 (210 - 610) | 45300 (40700 - 50300) | 2860 (2560 - 3170) |
| 1995 | 5590 (4170 - 6570) | 404 (246 - 695) | 20500 (18300 - 23000) | 2310 (2070 - 2570) | 4560 (2410 - 6520) | 237 (145 - 382) | 25600 (22800 - 28700) | 1280 (1150 - 1420) | 10100 (7570 - 12600) | 641 (395 - 1060) | 46200 (41300 - 51300) | 3590 (3220 - 3990) |
| 1996 | 5650 (4160 - 6670) | 627 (394 - 1030) | 21200 (18900 - 23700) | 2850 (2560 - 3170) | 4620 (2460 - 6610) | 383 (241 - 608) | 26200 (23300 - 29300) | 1670 (1500 - 1860) | 10300 (7680 - 12700) | 1010 (638 - 1650) | 47300 (42400 - 52600) | 4530 (4060 - 5030) |
| 1997 | 5730 (4190 - 6820) | 868 (559 - 1370) | 21900 (19600 - 24500) | 3540 (3160 - 3940) | 4640 (2500 - 6660) | 546 (353 - 851) | 26800 (23900 - 30100) | 2200 (1960 - 2450) | 10400 (7780 - 12800) | 1410 (921 - 2230) | 48800 (43700 - 54300) | 5730 (5120 - 6390) |
| 1998 | 5820 (4190 - 6960) | 1100 (729 - 1670) | 22800 (20400 - 25500) | 4240 (3780 - 4740) | 4680 (2550 - 6670) | 710 (463 - 1070) | 27600 (24500 - 30900) | 2750 (2460 - 3070) | 10500 (7890 - 13000) | 1810 (1210 - 2720) | 50400 (44900 - 56300) | 6990 (6240 - 7820) |
| 1999 | 5840 (4190 - 7020) | 1300 (867 - 1940) | 23700 (21100 - 26500) | 4840 (4310 - 5440) | 4660 (2620 - 6620) | 855 (555 - 1260) | 28300 (25100 - 31800) | 3240 (2880 - 3630) | 10500 (7820 - 13100) | 2150 (1440 - 3160) | 51900 (46200 - 58300) | 8080 (7190 - 9080) |
| 2000 | 5840 (4140 - 7090) | 1450 (946 - 2100) | 24400 (21500 - 27400) | 5190 (4620 - 5840) | 4620 (2610 - 6610) | 977 (636 - 1440) | 28900 (25500 - 32600) | 3540 (3150 - 3980) | 10500 (7750 - 13000) | 2430 (1590 - 3470) | 53300 (47400 - 59900) | 8730 (7770 - 9820) |
| 2001 | 5720 (4030 - 6960) | 1530 (944 - 2210) | 24800 (22100 - 27900) | 5300 (4720 - 5940) | 4500 (2580 - 6420) | 1050 (655 - 1540) | 29300 (25900 - 32900) | 3680 (3280 - 4130) | 10200 (7610 - 12800) | 2580 (1600 - 3630) | 54100 (48100 - 60600) | 8990 (7990 - 10100) |
| 2002 | 5540 (3880 - 6790) | 1530 (919 - 2230) | 25100 (22300 - 28300) | 5320 (4700 - 5960) | 4320 (2530 - 6350) | 1070 (651 - 1570) | 29300 (25900 - 33100) | 3780 (3340 - 4240) | 9860 (7340 - 12400) | 2600 (1570 - 3690) | 54400 (48100 - 61000) | 9100 (8040 - 10200) |
| 2003 | 5330 (3670 - 6560) | 1470 (867 - 2170) | 25300 (22300 - 28700) | 5260 (4650 - 5930) | 4100 (2390 - 6110) | 1050 (625 - 1590) | 29200 (25700 - 32900) | 3830 (3390 - 4330) | 9420 (7000 - 11900) | 2530 (1490 - 3700) | 54500 (48100 - 61400) | 9090 (8040 - 10300) |
| 2004 | 5140 (3530 - 6430) | 1370 (799 - 2040) | 25500 (22400 - 28900) | 5170 (4560 - 5830) | 3880 (2280 - 5930) | 1000 (584 - 1520) | 28900 (25500 - 32400) | 3850 (3390 - 4350) | 9020 (6710 - 11500) | 2370 (1380 - 3540) | 54400 (47800 - 61300) | 9020 (7950 - 10200) |
| 2005 | 4970 (3380 - 6280) | 1250 (718 - 1870) | 25700 (22600 - 29200) | 5100 (4480 - 5760) | 3680 (2130 - 5660) | 913 (528 - 1400) | 28600 (25100 - 32200) | 3850 (3380 - 4350) | 8650 (6430 - 11100) | 2160 (1250 - 3250) | 54300 (47700 - 61300) | 8950 (7850 - 10100) |
| 2006 | 4820 (3300 - 6110) | 1110 (640 - 1680) | 26000 (22800 - 29700) | 4880 (4280 - 5520) | 3500 (2050 - 5400) | 807 (470 - 1240) | 28000 (24600 - 31500) | 3720 (3270 - 4210) | 8310 (6220 - 10700) | 1910 (1110 - 2870) | 54000 (47300 - 61200) | 8600 (7550 - 9730) |
| 2007 | 4750 (3270 - 6020) | 964 (566 - 1470) | 26200 (23000 - 29800) | 4400 (3880 - 4970) | 3360 (2010 - 5200) | 687 (406 - 1050) | 27000 (23700 - 30500) | 3420 (3020 - 3860) | 8110 (6050 - 10400) | 1650 (971 - 2450) | 53200 (46700 - 60200) | 7820 (6890 - 8830) |
| 2008 | 4710 (3220 - 5940) | 845 (504 - 1270) | 26300 (23100 - 29900) | 3790 (3330 - 4280) | 3250 (1970 - 5000) | 574 (346 - 867) | 25800 (22600 - 29200) | 3040 (2670 - 3430) | 7950 (5970 - 10200) | 1420 (852 - 2060) | 52100 (45900 - 59000) | 6820 (6010 - 7710) |
| 2009 | 4680 (3220 - 5910) | 749 (458 - 1100) | 26500 (23200 - 30100) | 3190 (2810 - 3610) | 3150 (1940 - 4790) | 487 (301 - 733) | 24700 (21600 - 28000) | 2660 (2340 - 3000) | 7830 (5960 - 9940) | 1240 (761 - 1780) | 51200 (45000 - 57900) | 5850 (5140 - 6610) |
| 2010 | 4650 (3240 - 5860) | 665 (414 - 963) | 26600 (23300 - 30300) | 2790 (2440 - 3160) | 3090 (1950 - 4620) | 418 (263 - 625) | 24000 (20900 - 27300) | 2400 (2100 - 2720) | 7740 (5910 - 9810) | 1080 (678 - 1540) | 50600 (44400 - 57400) | 5180 (4540 - 5880) |
| 2011 | 4560 (3180 - 5750) | 587 (369 - 842) | 26700 (23500 - 30400) | 2550 (2230 - 2900) | 2990 (1880 - 4500) | 356 (223 - 535) | 23600 (20600 - 26900) | 2240 (1970 - 2550) | 7550 (5720 - 9520) | 944 (602 - 1330) | 50300 (44000 - 57100) | 4790 (4200 - 5450) |
| 2012 | 4440 (3120 - 5610) | 524 (336 - 743) | 26600 (23400 - 30300) | 2330 (2060 - 2650) | 2910 (1830 - 4370) | 311 (198 - 466) | 23100 (20200 - 26300) | 2100 (1850 - 2390) | 7350 (5520 - 9420) | 835 (539 - 1180) | 49700 (43800 - 56500) | 4430 (3910 - 5050) |
| 2013 | 4320 (3030 - 5470) | 476 (304 - 670) | 26400 (23200 - 30200) | 2140 (1880 - 2450) | 2820 (1760 - 4210) | 281 (181 - 421) | 22700 (19900 - 25900) | 1980 (1740 - 2260) | 7130 (5350 - 9220) | 757 (492 - 1060) | 49100 (43100 - 56100) | 4120 (3620 - 4710) |
| 2014 | 4200 (2970 - 5380) | 436 (280 - 614) | 26200 (23000 - 30300) | 1980 (1740 - 2270) | 2730 (1710 - 4200) | 259 (168 - 386) | 22300 (19400 - 25600) | 1860 (1640 - 2140) | 6930 (5220 - 8960) | 695 (450 - 974) | 48500 (42600 - 55700) | 3840 (3370 - 4410) |
| 2015 | 4080 (2880 - 5260) | 401 (260 - 569) | 26100 (22700 - 30500) | 1830 (1600 - 2120) | 2650 (1670 - 4130) | 238 (155 - 353) | 22000 (18900 - 25400) | 1760 (1540 - 2040) | 6730 (5080 - 8750) | 639 (417 - 898) | 48100 (42000 - 55700) | 3590 (3140 - 4160) |
| 2016 | 3960 (2800 - 5140) | 371 (240 - 526) | 25900 (22700 - 29800) | 1710 (1490 - 1960) | 2570 (1610 - 4050) | 218 (141 - 323) | 21600 (18800 - 24700) | 1670 (1460 - 1920) | 6530 (4890 - 8570) | 589 (385 - 829) | 47400 (41500 - 54500) | 3380 (2950 - 3880) |
| 2017 | 3830 (2690 - 4960) | 339 (219 - 487) | 25800 (22400 - 29700) | 1600 (1400 - 1830) | 2490 (1550 - 3980) | 201 (131 - 299) | 21300 (18500 - 24500) | 1590 (1390 - 1820) | 6320 (4680 - 8290) | 540 (355 - 768) | 47100 (41100 - 53900) | 3200 (2790 - 3650) |
| 2018 | 3740 (2610 - 4900) | 308 (198 - 443) | 25900 (22600 - 29900) | 1510 (1320 - 1740) | 2430 (1490 - 3980) | 186 (120 - 275) | 21300 (18600 - 24400) | 1520 (1330 - 1750) | 6170 (4510 - 8150) | 494 (322 - 700) | 47300 (41300 - 54400) | 3030 (2650 - 3480) |
| 2019 | 3660 (2520 - 4820) | 282 (181 - 409) | 25900 (22600 - 30100) | 1430 (1260 - 1660) | 2380 (1460 - 3910) | 174 (113 - 258) | 21200 (18200 - 24500) | 1460 (1280 - 1690) | 6050 (4410 - 8100) | 457 (295 - 649) | 47100 (41300 - 54500) | 2890 (2530 - 3340) |

Table S6. Tuberculosis deaths attributable and age-standardized population attributable fractions to smoking, alcohol use, and diabetes among individuals without HIV coinfection in Cambodia, 1990–2019.

|  | **Alcohol Use** | | **Diabetes** | | **Smoking** | |
| --- | --- | --- | --- | --- | --- | --- |
| **Year** | **Deaths** | **PAF** | **Deaths** | **PAF** | **Deaths** | **PAF** |
| 1990 | 513 (172 - 960) | 5.9 (1.9 - 11.5) | 370 (194 - 573) | 5.2 (2.9 - 8) | 1930 (1460 - 2400) | 25.8 (19.7 - 32.6) |
| 1991 | 605 (223 - 1090) | 6.9 (2.5 - 12.8) | 380 (200 - 589) | 5.3 (3 - 8.1) | 1950 (1480 - 2420) | 25.8 (19.8 - 32.4) |
| 1992 | 697 (266 - 1210) | 7.8 (3 - 14) | 392 (204 - 606) | 5.4 (3 - 8.2) | 1990 (1520 - 2450) | 25.7 (19.8 - 32.4) |
| 1993 | 794 (319 - 1360) | 8.7 (3.5 - 15.3) | 408 (213 - 629) | 5.5 (3.1 - 8.3) | 2040 (1560 - 2520) | 25.7 (19.7 - 32.3) |
| 1994 | 892 (370 - 1520) | 9.5 (3.9 - 16.3) | 429 (223 - 658) | 5.5 (3.1 - 8.4) | 2100 (1610 - 2600) | 25.7 (19.9 - 32.2) |
| 1995 | 987 (432 - 1640) | 10.3 (4.4 - 17.3) | 449 (235 - 688) | 5.7 (3.2 - 8.6) | 2170 (1660 - 2690) | 25.8 (19.8 - 32.3) |
| 1996 | 1070 (493 - 1730) | 10.9 (4.8 - 18.2) | 469 (248 - 721) | 5.8 (3.3 - 8.8) | 2220 (1680 - 2770) | 25.8 (20 - 32.4) |
| 1997 | 1160 (569 - 1840) | 11.6 (5.3 - 18.9) | 493 (262 - 758) | 6 (3.4 - 9.1) | 2290 (1720 - 2850) | 26.1 (20.1 - 32.6) |
| 1998 | 1240 (613 - 1960) | 12.2 (5.7 - 19.9) | 520 (278 - 807) | 6.2 (3.5 - 9.4) | 2370 (1740 - 2950) | 26.4 (20.1 - 32.8) |
| 1999 | 1320 (652 - 2070) | 12.8 (6 - 20.6) | 541 (290 - 839) | 6.4 (3.6 - 9.7) | 2420 (1770 - 3030) | 26.7 (20.2 - 33.3) |
| 2000 | 1380 (682 - 2180) | 13.4 (6.4 - 21.3) | 559 (299 - 873) | 6.6 (3.7 - 10) | 2460 (1790 - 3100) | 26.9 (20.4 - 33.6) |
| 2001 | 1410 (709 - 2200) | 14 (6.9 - 21.9) | 563 (299 - 874) | 6.7 (3.8 - 10.3) | 2440 (1780 - 3090) | 27.2 (20.6 - 33.7) |
| 2002 | 1420 (732 - 2190) | 14.4 (7.2 - 22.5) | 558 (293 - 870) | 6.9 (3.8 - 10.6) | 2390 (1750 - 3050) | 27.4 (20.8 - 34) |
| 2003 | 1410 (742 - 2190) | 15 (7.5 - 23) | 550 (284 - 851) | 7 (4 - 10.8) | 2330 (1690 - 2980) | 27.7 (21 - 34.3) |
| 2004 | 1420 (755 - 2220) | 15.6 (7.9 - 23.6) | 544 (288 - 836) | 7.2 (4 - 11.1) | 2270 (1640 - 2960) | 28 (21.3 - 34.6) |
| 2005 | 1430 (763 - 2230) | 16.3 (8.5 - 24.5) | 539 (286 - 831) | 7.4 (4.1 - 11.3) | 2210 (1580 - 2920) | 28.2 (21.3 - 35) |
| 2006 | 1460 (791 - 2260) | 17.1 (9.2 - 25.4) | 540 (288 - 832) | 7.7 (4.3 - 11.8) | 2150 (1530 - 2840) | 28.3 (21.5 - 35) |
| 2007 | 1520 (857 - 2330) | 18.3 (10.1 - 26.7) | 548 (289 - 843) | 7.9 (4.4 - 12.1) | 2120 (1500 - 2810) | 28.4 (21.6 - 35) |
| 2008 | 1600 (918 - 2410) | 19.5 (11.2 - 28.1) | 560 (297 - 865) | 8.2 (4.5 - 12.5) | 2100 (1480 - 2780) | 28.5 (21.7 - 34.9) |
| 2009 | 1680 (996 - 2480) | 20.7 (12 - 29.5) | 577 (309 - 894) | 8.5 (4.7 - 13) | 2090 (1490 - 2730) | 28.6 (21.9 - 34.8) |
| 2010 | 1750 (1050 - 2560) | 21.8 (12.9 - 30.9) | 595 (321 - 914) | 8.8 (4.9 - 13.5) | 2070 (1480 - 2690) | 28.5 (22 - 34.8) |
| 2011 | 1800 (1100 - 2600) | 22.9 (13.9 - 31.8) | 609 (329 - 942) | 9.2 (5.1 - 14) | 2030 (1450 - 2640) | 28.5 (21.8 - 34.8) |
| 2012 | 1840 (1120 - 2640) | 23.9 (14.9 - 32.9) | 621 (340 - 965) | 9.5 (5.3 - 14.7) | 1980 (1420 - 2590) | 28.4 (21.7 - 34.6) |
| 2013 | 1870 (1150 - 2650) | 25 (16 - 33.9) | 633 (342 - 990) | 10 (5.5 - 15.2) | 1920 (1370 - 2520) | 28.3 (21.5 - 34.4) |
| 2014 | 1880 (1170 - 2680) | 25.9 (16.8 - 35.1) | 643 (344 - 1000) | 10.4 (5.8 - 15.8) | 1870 (1330 - 2450) | 28.1 (21.4 - 34.3) |
| 2015 | 1880 (1180 - 2650) | 26.7 (17.5 - 36.2) | 653 (339 - 1020) | 10.8 (6 - 16.5) | 1810 (1270 - 2390) | 27.9 (21.2 - 34) |
| 2016 | 1880 (1180 - 2650) | 27.4 (18 - 36.7) | 661 (344 - 1030) | 11.2 (6.3 - 17) | 1740 (1230 - 2330) | 27.6 (21 - 33.8) |
| 2017 | 1840 (1170 - 2620) | 27.8 (18.1 - 37.3) | 669 (351 - 1060) | 11.6 (6.5 - 17.5) | 1680 (1190 - 2280) | 27.4 (20.7 - 33.7) |
| 2018 | 1820 (1160 - 2620) | 28 (18.2 - 37.6) | 683 (363 - 1080) | 12 (6.8 - 18.2) | 1640 (1150 - 2240) | 27.2 (20.5 - 33.5) |
| 2019 | 1790 (1120 - 2600) | 28.1 (18.2 - 37.9) | 699 (375 - 1100) | 12.5 (7.1 - 19) | 1600 (1110 - 2210) | 27 (20.2 - 33.3) |

Table S7. Tuberculosis age-standardized population attributable fractions to smoking, alcohol use, and diabetes among males and females without HIV coinfection in Cambodia, 1990–2019

|  | **Alcohol** | | **Diabetes** | | **Smoking** | |
| --- | --- | --- | --- | --- | --- | --- |
| **Year** | **Male** | **Female** | **Male** | **Female** | **Male** | **Female** |
| 1990 | 9.0 (3.3 - 16.1) | 1.2 (0.1 - 2.6) | 4.2 (2.5 - 6.2) | 3.7 (2.2 - 5.4) | 33.7 (28.8 - 38.3) | 4.6 (3.0 - 6.2) |
| 1991 | 10.5 (4.4 - 18.0) | 1.5 (0.2 - 3.1) | 4.2 (2.5 - 6.3) | 3.8 (2.3 - 5.5) | 33.7 (28.7 - 38.4) | 4.7 (3.1 - 6.3) |
| 1992 | 11.9 (5.3 - 19.8) | 1.7 (0.3 - 3.6) | 4.3 (2.5 - 6.4) | 3.8 (2.3 - 5.6) | 33.8 (28.8 - 38.5) | 4.7 (3.1 - 6.3) |
| 1993 | 13.2 (6.3 - 21.7) | 1.9 (0.4 - 4.0) | 4.4 (2.6 - 6.7) | 3.9 (2.3 - 5.8) | 34.1 (29.1 - 38.8) | 4.8 (3.2 - 6.4) |
| 1994 | 14.5 (7.0 - 23.4) | 2.2 (0.5 - 4.4) | 4.5 (2.6 - 6.9) | 4.0 (2.4 - 6.0) | 34.4 (29.3 - 39.0) | 4.9 (3.3 - 6.5) |
| 1995 | 15.7 (7.8 - 24.9) | 2.4 (0.6 - 4.8) | 4.7 (2.7 - 7.0) | 4.1 (2.5 - 6.2) | 34.7 (29.5 - 39.3) | 5.0 (3.4 - 6.6) |
| 1996 | 16.8 (8.6 - 25.7) | 2.6 (0.8 - 5.1) | 4.8 (2.8 - 7.2) | 4.3 (2.6 - 6.4) | 35.1 (29.9 - 39.7) | 5.1 (3.5 - 6.7) |
| 1997 | 17.9 (9.6 - 26.9) | 2.9 (0.8 - 5.5) | 5.0 (2.9 - 7.4) | 4.4 (2.7 - 6.6) | 35.7 (30.3 - 40.3) | 5.3 (3.6 - 6.9) |
| 1998 | 18.9 (10.4 - 27.9) | 3.1 (1.0 - 5.8) | 5.2 (3.1 - 7.8) | 4.6 (2.8 - 6.9) | 36.3 (30.9 - 41.1) | 5.4 (3.8 - 7.1) |
| 1999 | 19.9 (11.1 - 29.2) | 3.3 (1.1 - 6.1) | 5.5 (3.2 - 8.1) | 4.8 (2.9 - 7.1) | 36.9 (31.9 - 41.5) | 5.6 (3.9 - 7.3) |
| 2000 | 20.9 (11.8 - 30.5) | 3.5 (1.2 - 6.4) | 5.7 (3.4 - 8.4) | 4.9 (3.0 - 7.4) | 37.5 (32.3 - 42.3) | 5.8 (4.0 - 7.5) |
| 2001 | 21.8 (12.4 - 31.3) | 3.7 (1.3 - 6.8) | 5.9 (3.5 - 8.7) | 5.0 (3.0 - 7.6) | 38.0 (32.8 - 42.8) | 5.9 (4.2 - 7.6) |
| 2002 | 22.6 (13.0 - 32.1) | 3.9 (1.4 - 7.0) | 6.1 (3.6 - 9.0) | 5.1 (3.1 - 7.7) | 38.4 (33.1 - 43.4) | 6.0 (4.3 - 7.8) |
| 2003 | 23.4 (13.8 - 32.8) | 4.1 (1.5 - 7.3) | 6.3 (3.7 - 9.3) | 5.3 (3.1 - 7.8) | 38.9 (33.4 - 43.9) | 6.2 (4.4 - 8.0) |
| 2004 | 24.3 (14.5 - 34.0) | 4.3 (1.6 - 7.6) | 6.5 (3.8 - 9.6) | 5.4 (3.2 - 8.1) | 39.3 (33.8 - 44.3) | 6.3 (4.5 - 8.2) |
| 2005 | 25.3 (15.2 - 35.2) | 4.6 (1.7 - 8.0) | 6.7 (3.9 - 9.9) | 5.6 (3.3 - 8.4) | 39.6 (34.1 - 44.7) | 6.5 (4.6 - 8.3) |
| 2006 | 26.6 (16.3 - 36.4) | 5.0 (2.0 - 8.5) | 7.0 (4.1 - 10.2) | 5.9 (3.4 - 8.7) | 39.9 (34.4 - 45.0) | 6.5 (4.6 - 8.4) |
| 2007 | 28.2 (17.9 - 37.8) | 5.5 (2.4 - 9.1) | 7.2 (4.2 - 10.6) | 6.1 (3.6 - 9.1) | 40.0 (34.5 - 45.1) | 6.5 (4.6 - 8.3) |
| 2008 | 29.9 (19.6 - 39.6) | 6.0 (2.6 - 9.8) | 7.4 (4.4 - 11.0) | 6.5 (3.8 - 9.6) | 40.0 (34.5 - 45.3) | 6.5 (4.7 - 8.3) |
| 2009 | 31.5 (21.0 - 41.4) | 6.5 (2.9 - 10.7) | 7.7 (4.6 - 11.5) | 6.8 (4.0 - 10.2) | 40.1 (34.7 - 45.4) | 6.5 (4.7 - 8.4) |
| 2010 | 33.0 (22.1 - 42.9) | 7.1 (3.3 - 11.6) | 8.0 (4.8 - 11.9) | 7.2 (4.1 - 10.7) | 40.2 (34.6 - 45.6) | 6.5 (4.6 - 8.4) |
| 2011 | 34.5 (23.3 - 44.4) | 7.7 (3.8 - 12.5) | 8.4 (5.0 - 12.4) | 7.5 (4.3 - 11.2) | 40.4 (34.7 - 45.8) | 6.5 (4.6 - 8.3) |
| 2012 | 35.9 (24.9 - 45.7) | 8.4 (4.2 - 13.4) | 8.8 (5.2 - 12.9) | 7.9 (4.6 - 11.9) | 40.4 (34.6 - 45.9) | 6.4 (4.5 - 8.2) |
| 2013 | 37.3 (26.5 - 47.2) | 9.1 (4.8 - 14.3) | 9.2 (5.4 - 13.5) | 8.3 (4.8 - 12.6) | 40.4 (34.6 - 46.0) | 6.4 (4.5 - 8.1) |
| 2014 | 38.5 (27.4 - 48.3) | 9.8 (5.2 - 15.4) | 9.7 (5.6 - 14.3) | 8.7 (5.0 - 13.3) | 40.3 (34.4 - 46.0) | 6.3 (4.4 - 8.1) |
| 2015 | 39.5 (28.1 - 49.4) | 10.4 (5.5 - 16.1) | 10.1 (5.9 - 15.0) | 9.2 (5.3 - 14.0) | 40.2 (34.3 - 46.0) | 6.2 (4.4 - 8.0) |
| 2016 | 40.3 (29.1 - 50.3) | 10.9 (5.7 - 16.9) | 10.5 (6.2 - 15.5) | 9.6 (5.5 - 14.5) | 40.0 (34.1 - 45.9) | 6.2 (4.3 - 8.1) |
| 2017 | 40.9 (29.2 - 51.0) | 11.2 (5.9 - 17.5) | 11.0 (6.5 - 16.1) | 10.0 (5.8 - 15.2) | 39.9 (34.0 - 45.9) | 6.1 (4.2 - 8.0) |
| 2018 | 41.2 (29.2 - 51.4) | 11.4 (5.9 - 17.7) | 11.4 (6.8 - 16.8) | 10.5 (6.1 - 15.7) | 39.8 (33.8 - 45.8) | 6.0 (4.2 - 8.0) |
| 2019 | 41.4 (29.2 - 51.6) | 11.4 (5.9 - 17.8) | 11.9 (7.1 - 17.4) | 11.1 (6.5 - 16.6) | 39.8 (33.7 - 45.8) | 6.0 (4.1 - 7.9) |

Figure S5. Temporal trends of age-standardized tuberculosis mortality rate per 100,000 population (A) and deaths (B) in Cambodia by HIV status and sex, 1990–2019


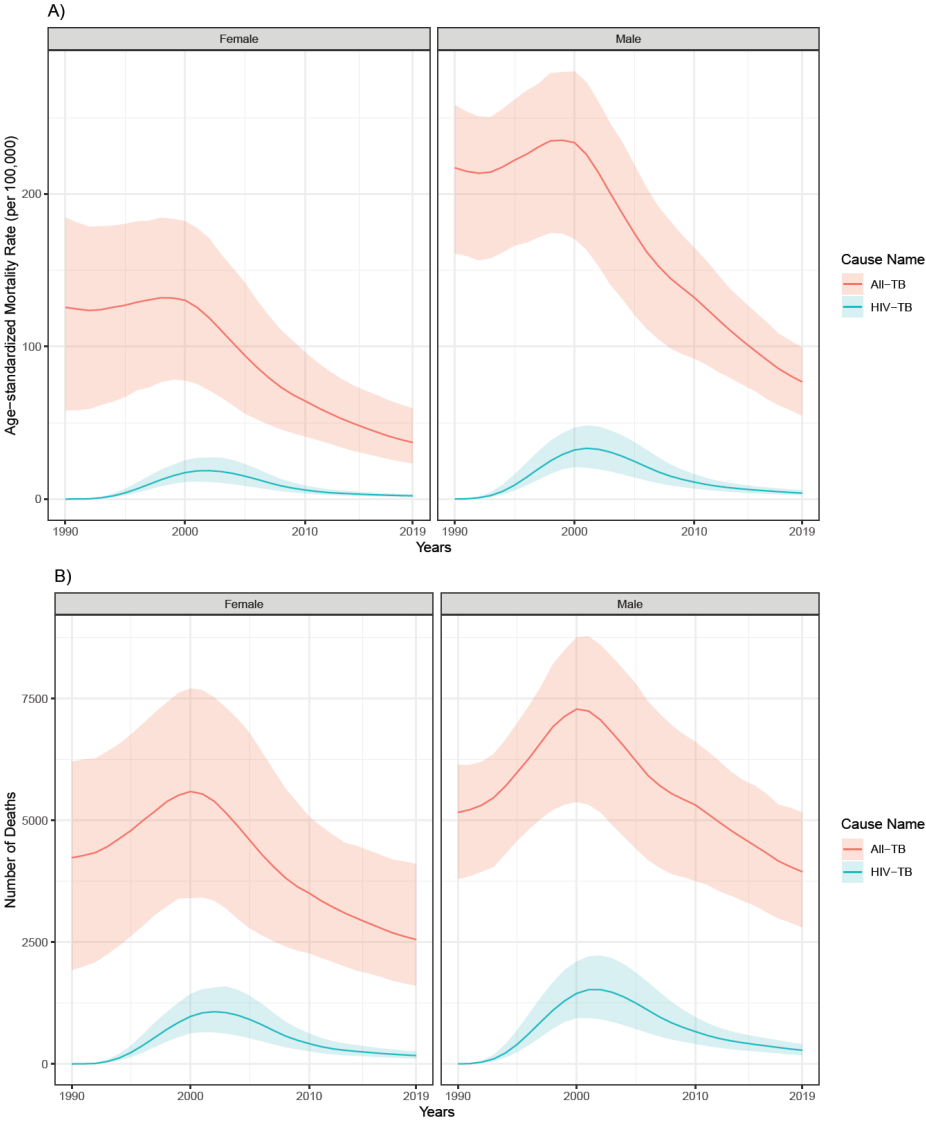


Figure S6. Temporal trends of age-standardized tuberculosis incidence rate per 100,000 population (A) and incident cases (B) in Cambodia by HIV status and sex, 1990–2019


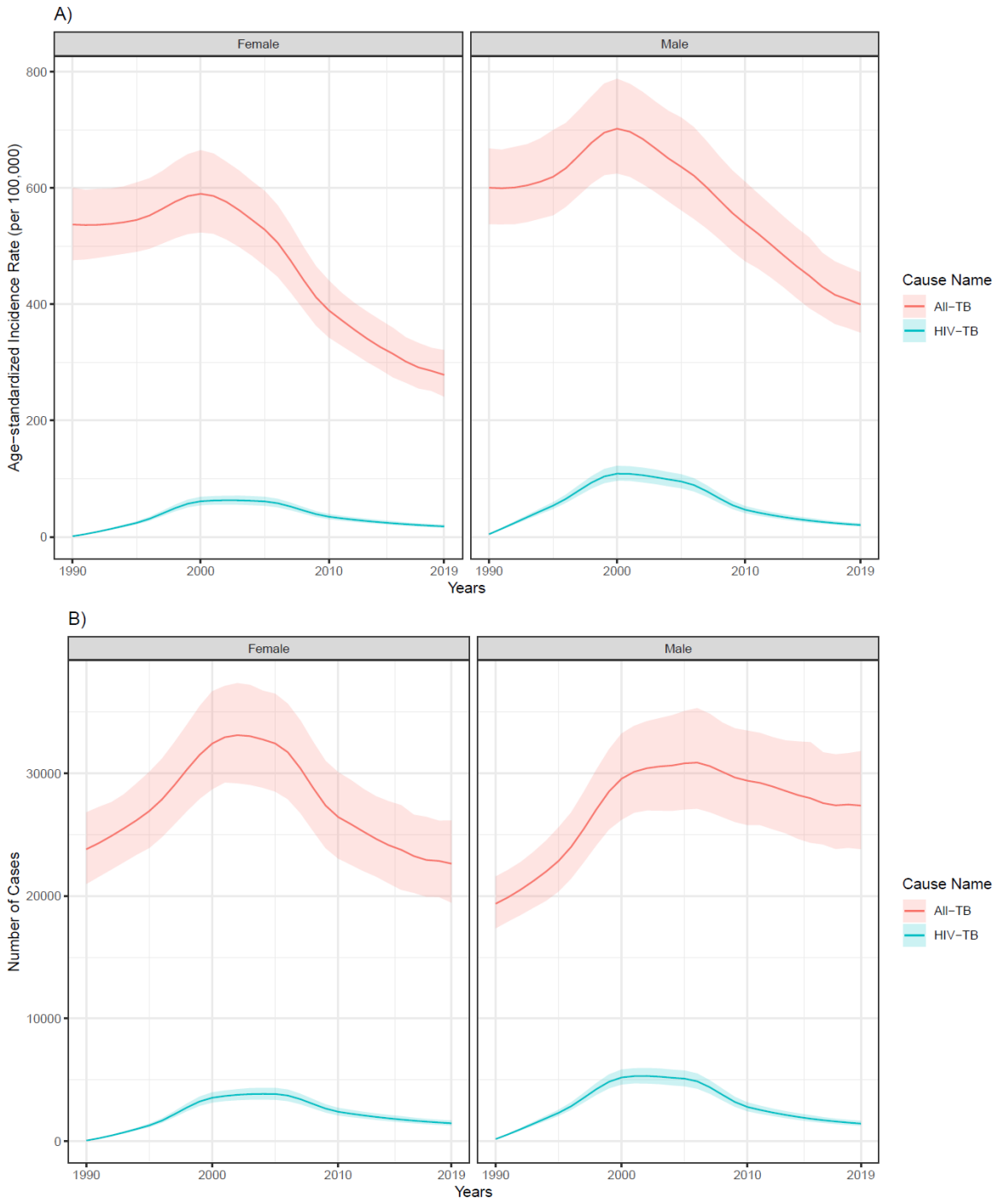


Figure S7. Age-standardized population attributable fractions of tuberculosis deaths due to alcohol use, smoking, and diabetes among individuals without HIV coinfection in Cambodia by year and sex


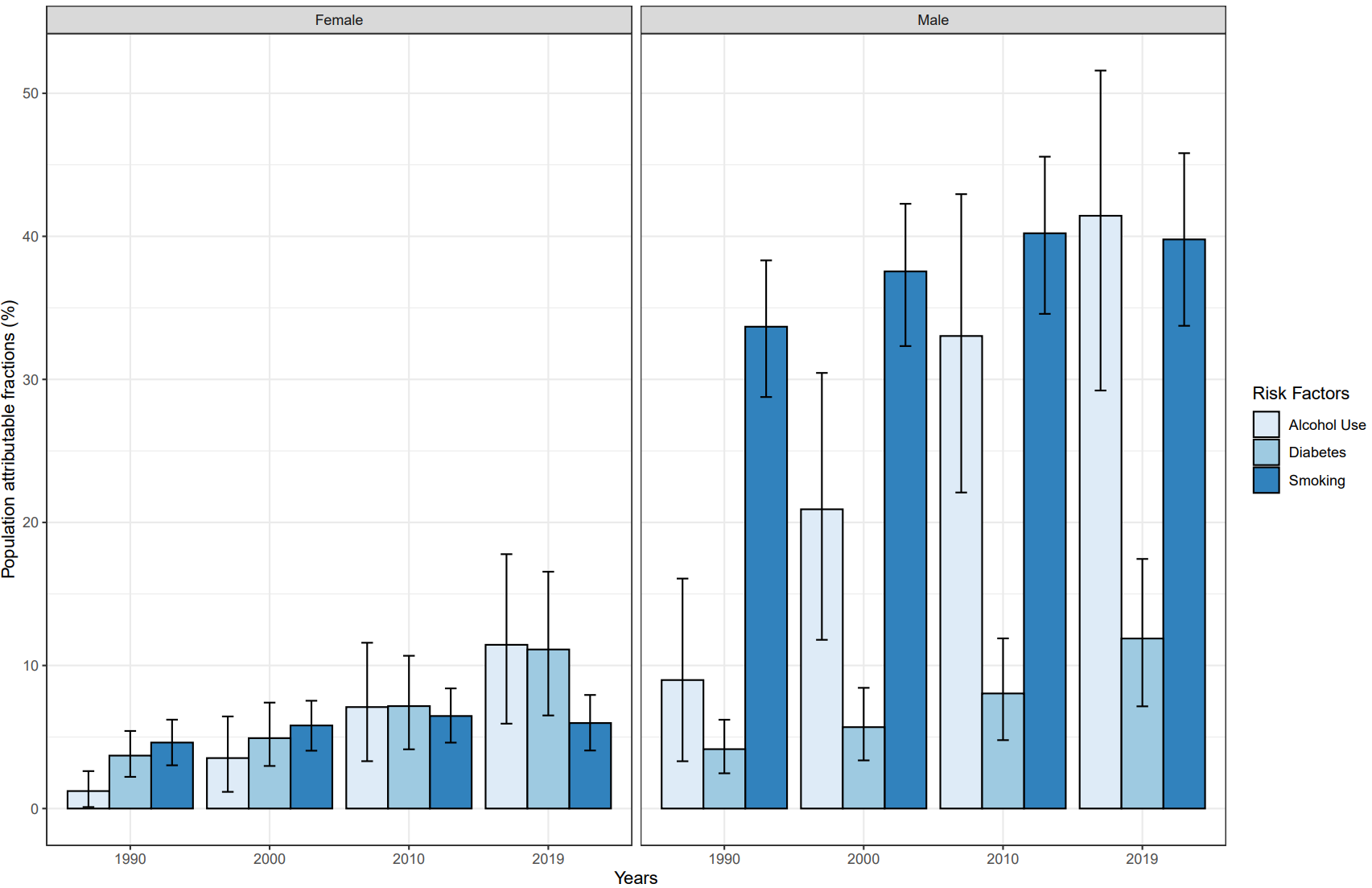


**References**

1. Vos T, Lim SS, Abbafati C, Abbas KM, Abbasi M, Abbasifard M, et al. Global burden of 369 diseases and injuries in 204 countries and territories, 1990–2019: a systematic analysis for the Global Burden of Disease Study 2019. The Lancet [Internet]. 2020 Oct;396(10258):1204–22. Available from: https://linkinghub.elsevier.com/retrieve/pii/S0140673620309259

2. Murray CJL, Aravkin AY, Zheng P, Abbafati C, Abbas KM, Abbasi-Kangevari M, et al. Global burden of 87 risk factors in 204 countries and territories, 1990–2019: a systematic analysis for the Global Burden of Disease Study 2019. The Lancet [Internet]. 2020 Oct;396(10258):1223–49. Available from: https://linkinghub.elsevier.com/retrieve/pii/S0140673620307522

3. Kyu HH, Maddison ER, Henry NJ, Mumford JE, Barber R, Shields C, et al. The global burden of tuberculosis: results from the Global Burden of Disease Study 2015. The Lancet Infectious Diseases [Internet]. 2018 Mar;18(3):261–84. Available from: https://linkinghub.elsevier.com/retrieve/pii/S147330991730703X

4. Kyu HH, Maddison ER, Henry NJ, Ledesma JR, Wiens KE, Reiner R, et al. Global, regional, and national burden of tuberculosis, 1990–2016: results from the Global Burden of Diseases, Injuries, and Risk Factors 2016 Study. The Lancet Infectious Diseases [Internet]. 2018 Dec;18(12):1329–49. Available from: https://linkinghub.elsevier.com/retrieve/pii/S147330991830625X

5. Ledesma JR, Ma J, Vongpradith A, Maddison ER, Novotney A, Biehl MH, et al. Global, regional, and national sex differences in the global burden of tuberculosis by HIV status, 1990–2019: results from the Global Burden of Disease Study 2019. The Lancet Infectious Diseases. 2022 Feb;22(2):222–41.

6. Naghavi M, Makela S, Foreman K, O’Brien J, Pourmalek F, Lozano R. Algorithms for enhancing public health utility of national causes-of-death data. Population Health Metrics [Internet]. 2010 Dec 10;8(1):9. Available from: https://pophealthmetrics.biomedcentral.com/articles/10.1186/1478-7954-8-9

7. Adegbola RA, Falade AG, Sam BE, Aidoo M, Baldeh I, Hazlett D, et al. The etiology of pneumonia in malnourished and well-nourished Gambian children. The Pediatric infectious disease journal. 1994 Nov;13(11):975–82.

8. Chisti MJ, Graham SM, Duke T, Ahmed T, Ashraf H, Faruque ASG, et al. A prospective study of the prevalence of tuberculosis and bacteraemia in Bangladeshi children with severe malnutrition and pneumonia including an evaluation of Xpert MTB/RIF assay. PloS one. 2014;9(4):e93776.

9. Madhi SA, Petersen K, Madhi A, Khoosal M, Klugman KP. Increased disease burden and antibiotic resistance of bacteria causing severe community-acquired lower respiratory tract infections in human immunodeficiency virus type 1-infected children. Clinical infectious diseases : an official publication of the Infectious Diseases Society of America. 2000 Jul;31(1):170–6.

10. McNally LM, Jeena PM, Gajee K, Thula SA, Sturm AW, Cassol S, et al. Effect of age, polymicrobial disease, and maternal HIV status on treatment response and cause of severe pneumonia in South African children: a prospective descriptive study. The Lancet. 2007 Apr;369(9571):1440–51.

11. Moore DP, Klugman KP, Madhi SA. Role of streptococcus pneumoniae in hospitalization for acute community-acquired pneumonia associated with culture-confirmed mycobacterium tuberculosis in Children. The Pediatric Infectious Disease Journal. 2010 Dec;29(12):1099–104.

12. Nantongo JM, Wobudeya E, Mupere E, Joloba M, Ssengooba W, Kisembo HN, et al. High incidence of pulmonary tuberculosis in children admitted with severe pneumonia in Uganda. BMC Pediatrics. 2013 Dec 31;13(1):16.

13. Zar HJ, Hanslo D, Tannenbaum E, Klein M, Argent A, Eley B, et al. Aetiology and outcome of pneumonia in human immunodeficiency virus-infected children hospitalized in South Africa. Acta paediatrica. 2001 Feb;90(2):119–25.

14. Moore DP, Higdon MM, Hammitt LL, Prosperi C, DeLuca AN, Da Silva P, et al. The incremental value of repeated induced sputum and gastric aspirate samples for the diagnosis of pulmonary tuberculosis in young children with acute community-acquired pneumonia. Clinical Infectious Diseases. 2017 Jun 15;64(suppl_3):S309–16.

15. Foreman KJ, Lozano R, Lopez AD, Murray CJ. Modeling causes of death: an integrated approach using CODEm. Population Health Metrics. 2012 Dec 6;10(1):1.

16. Cox JA, Lukande RL, Lucas S, Nelson AM, Van Marck E, Colebunders R. Autopsy causes of death in HIV-positive individuals in sub-Saharan Africa and correlation with clinical diagnoses. AIDS reviews. 2010;12(4):183–94.

17. Ford N, Matteelli A, Shubber Z, Hermans S, Meintjes G, Grinsztejn B, et al. TB as a cause of hospitalization and in-hospital mortality among people living with HIV worldwide: a systematic review and meta-analysis. Journal of the International AIDS Society. 2016 Jan;19(1):20714.

18. Zheng P, Barber R, Sorensen RJD, Murray CJL, Aravkin AY. Trimmed constrained mixed effects models: formulations and algorithms. Journal of Computational and Graphical Statistics [Internet]. 2021 Jul 3;30(3):544–56. Available from: https://www.tandfonline.com/doi/full/10.1080/10618600.2020.1868303

19. Fullman N, Yearwood J, Abay SM, Abbafati C, Abd-Allah F, Abdela J, et al. Measuring performance on the Healthcare Access and Quality Index for 195 countries and territories and selected subnational locations: a systematic analysis from the Global Burden of Disease Study 2016. The Lancet. 2018 Jun;391(10136):2236–71.

20. National Tuberculosis Institute. Tuberculosis in a rural population of South India: a five-year epidemiological study. Bulletin of the World Health Organization. 1974;51(5):473–88.

21. Japan International Cooperation Agency, National Center for Tuberculosis and Leprosy Control (CENAT) (Cambodia) RI of TA-TA (RIT/JATA). Cambodia National Tuberculosis Prevalence Survey 2002.

22. Japan International Cooperation Agency, Ministry of Health (Cambodia), National Center for Tuberculosis and Leprosy Control (CENAT) (Cambodia), Research Institute of Tuberculosis/Japan Anti-Tuberculosis Association (RIT/JATA) WHO (WHO). Cambodia National Tuberculosis Prevalence Survey 2010-2011.

23. Chinese Center for Disease Control and Prevention (CCDC), Ministry of Health (China) WHO (WHO). China National Tuberculosis Prevalence Survey 2010.

24. Chinese Center for Disease Control and Prevention. China National Tuberculosis Prevalence Survey 2000.

25. Ministry of Public Health (China), National Tuberculosis Control and Research Center, Beijing (China), National Tuberculosis Control and Research Subcenter S (China). China National Tuberculosis Prevalence Survey 1984-1985.

26. Ethiopian Health and Nutrition Research Center (EHNRI), Ministry of Health (Ethiopia) WHO (WHO). Ethiopia Tuberculosis Prevalence Survey 2010-2011.

27. (China) M of PH. China National Tuberculosis Prevalence Survey 1990.

28. Tupasi TE, Radhakrishna S, Chua JA, Mangubat N V, Guilatco R, Galipot M, et al. Significant decline in the tuberculosis burden in the Philippines ten years after initiating DOTS. The International Journal of Tuberculosis and Lung Disease. 2009 Oct;13(10):1224–30.

29. Hoa NB, Sy DN, Nhung NV, Tiemersma EW, Borgdorff MW, Cobelens FG. National survey of tuberculosis prevalence in Viet Nam. Bulletin of the World Health Organization. 2010 Apr 1;88(4):273–80.

30. Central TB Division, Directorate General of Health Services (India), Ministry of Health and Family Welfare (India). Delhi, India: Central TB Division DG of HS (India). India Revised National Tuberculosis Control Program Annual Report 2013. 2013.

31. Ministry of Health (Myanmar), Research Institute of Tuberculosis/Japan Anti-Tuberculosis Association (RIT/JATA) WHO (WHO). Myanmar National Tuberculosis Prevalence Survey 2009-2010.

32. Hong YP, Kim SJ, Lew WJ, Lee EK, Han YC. The seventh nationwide tuberculosis prevalence survey in Korea, 1995. The International Journal of Tuberculosis and Lung Disease. 1998 Jan;2(1):27–36.

33. Centers for Disease Control and Prevention (CDC), KNCV Tuberculosis Foundation, Ministry of Health (Rwanda), National TB Control Program (Rwanda), Rwanda Biomedical Center (RBC), University Teaching Hospital of Kigali (CHUK) WHO (WHO). Rwanda National Tuberculosis Prevalence Survey 2012.

34. Datta M, Radhamani MP, Sadacharam K, Selvaraj R, Rao DL, Rao RS, et al. Survey for tuberculosis in a tribal population in North Arcot District. The International Journal of Tuberculosis and Lung Disease. 2001 Mar;5(3):240–9.

35. National Leprosy and Tuberculosis Control Program (NTLP) (The Gambia) MRC (Gambia). Gambia National Tuberculosis Prevalence Survey 2011-2013 (GAMSTEP).

36. Berhe G, Enqueselassie F, Hailu E, Mekonnen W, Teklu T, Gebretsadik A, et al. Population-based prevalence survey of tuberculosis in the Tigray region of Ethiopia. BMC Infectious Diseases. 2013 Dec 28;13(1):448.

37. Health and Family Welfare Department Government of Gujarat. Population based survey for assessing prevalence of pulmonary tuberculosis cases in the state of Gujarat, India (2011-2012). 2013.

38. Gothi G, Narayan R, Nair S, Chakraborty A, Srikantaramu N. Estimation of prevalence of bacillary tuberculosis on the basis of chest X-ray and/or symptomatic screening. Indian Journal of Medical Research. 1976;64(8):1150–9.

39. Chadha VK, Kumar P, Anjinappa SM, Singh S, Narasimhaiah S, Joshi M V., et al. Prevalence of Pulmonary Tuberculosis among Adults in a Rural Sub-District of South India. Pai M, editor. PLoS ONE. 2012 Aug 15;7(8):e42625.

40. Pg G, Sadacharam K, Narayanan P. Yield of pulmonary tuberculosis cases by employing two screening methods in a community survey. The international journal of tuberculosis and lung disease : the official journal of the International Union against Tuberculosis and Lung Disease. 2006 Apr 1;10:343–5.

41. Datta M, Pg G, Appegowda B, Rao K, Gopalan B. Tuberculosis in north Arcot district of Tamil Nadu – a sample survey. Indian Journal of Tuberculosis. 2000 Jan 1;47.

42. Tiemersma EW, van der Werf MJ, Borgdorff MW, Williams BG, Nagelkerke NJD. Natural history of tuberculosis: duration and fatality of untreated pulmonary tuberculosis in HIV negative patients: a systematic review. Pai M, editor. PLoS ONE. 2011 Apr 4;6(4):e17601.
